# United States Dietary Trends Since 1800: Lack of Association Between Saturated Fatty Acid Consumption and Non-communicable Diseases

**DOI:** 10.3389/fnut.2021.748847

**Published:** 2022-01-13

**Authors:** Joyce H. Lee, Miranda Duster, Timothy Roberts, Orrin Devinsky

**Affiliations:** ^1^Department of Neurology, New York University, Grossman School of Medicine, New York, NY, United States; ^2^Medical College of Wisconsin, Milwaukee, WI, United States; ^3^New York University, Health Sciences Library, New York, NY, United States

**Keywords:** obesity, diabetes, non-communicable diseases (NCDs), processed foods, nutrients

## Abstract

We reviewed data on the American diet from 1800 to 2019.

**Methods:** We examined food availability and estimated consumption data from 1800 to 2019 using historical sources from the federal government and additional public data sources.

**Results:** Processed and ultra-processed foods increased from <5 to >60% of foods. Large increases occurred for sugar, white and whole wheat flour, rice, poultry, eggs, vegetable oils, dairy products, and fresh vegetables. Saturated fats from animal sources declined while polyunsaturated fats from vegetable oils rose. Non-communicable diseases (NCDs) rose over the twentieth century in parallel with increased consumption of processed foods, including sugar, refined flour and rice, and vegetable oils. Saturated fats from animal sources were inversely correlated with the prevalence of NCDs.

**Conclusions:** As observed from the food availability data, processed and ultra-processed foods dramatically increased over the past two centuries, especially sugar, white flour, white rice, vegetable oils, and ready-to-eat meals. These changes paralleled the rising incidence of NCDs, while animal fat consumption was inversely correlated.

## Introduction

The American diet changed radically since 1800 due to industrial and technological advances, geographic spread, urbanization, wars, cultural changes, as well as food industry conglomerates and globalization. Foods became progressively more processed, associated with a parallel but delayed rise in non-communicable diseases (NCDs) in the United States, other western nations, and more recently, in developing nations, as their diets and lifestyles westernized. Chronic NCDs include metabolic syndrome [abdominal obesity, hypertension, non-alcoholic fatty liver disease, insulin resistance, hyperinsulinemia, elevated triglycerides, low high-density lipoprotein (HDL)], type 2 diabetes (T2D), gout, heart disease, stroke, cancer, polycystic ovarian syndrome, and Alzheimer's disease (1, 2).

Ancel Keys' Diet-Heart Hypothesis posited that the mid-nineteenth century heart disease epidemic resulted from “a changing American diet”: increased consumption of fats, especially saturated fatty acids (SFAs), and decreased grain consumption (3). Supporting evidence included (1) hypercholesterolemia in heart disease patients, (2) rabbits fed high fat diets developed hypercholesterolemia and atherosclerotic-like lesions, (3) cholesterol comprised 40–70% of atherosclerotic plaques, (4) SFAs increased serum cholesterol in short-term feeding studies, (5) high fat diets were associated with higher cholesterol levels in some populations, (6) fat consumption was correlated with heart disease deaths over time in the United States and six other countries, and (7) familial hypercholesterolemia patients had increased heart disease rates (4–7).

In 1961, the American Heart Association first recommended that men at high-risk for cardiac disease should reduce total fat consumption to 25–35% of calories and substitute polyunsaturated fatty acids (PUFAs) for SFAs (8). Neither prospective studies nor randomized trials supported that recommendation (9, 10). In 1977, Senator George McGovern's Dietary Goals transformed this hypothesis to national policy and extended SFA's effects to obesity and cancer and recommended reductions for everyone over age 2 years (11). The Surgeon General, National Research Council, and American Cancer Society also recommended low-fat or low-SFA diets to reduce coronary heart disease (CHD) and cancer (11–14). However, some studies correlated low cholesterol levels with higher cancer rates and low-fat diets or substituting PUFAs for SFAs correlated with increased mortality, cardiovascular disease, and cancer (15–20). Prospective studies and randomized trials found that dietary fat, SFAs, or elevated cholesterol levels were not associated with increased cancer risk (21, 22).

The belief that fat and SFAs drive obesity and heart disease has persisted more than a century (23). In 1960, the Framingham Heart Study found no link between fat or SFA consumption and heart disease, but this data was never published (9, 24). In 1967, Fredrickson (NIH Director 1975–1981), Levy (NHLBI Director 1975–1981), and Lees (Rockefeller University) wrote a five-part *New England Journal of Medicine* series that identified elevated very-low-density lipoprotein (VLDL) cholesterol as the most common lipoprotein disorder. A “sizable fraction of the population suffering from coronary heart disease” with “carbohydrate-induced hyperlipidemia” should be treated with “weight control, avoidance of excessive dietary carbohydrates and hypolipemic agents (25).” The identification of metabolic syndrome and its association in animal models and humans with diets rich in refined carbohydrates further supported that fats and SFA were not the primary dietary drivers of obesity and heart disease (26, 27).

A foundation of Keys' Diet-Heart Hypothesis and McGovern's *Dietary Goals* was that heart disease resulted from America's declining grain and increasing fat and SFA consumption (3, 11). Short-term studies do not replicate long-term metabolic and hormonal changes, and population-based studies over time are limited by reliability and validity (28–30). We reviewed the available data to examine the American diet from 1800 to 2019.

## Materials and Methods

We analyzed data from 1800 to 2019 on food availability for categories (e.g., red meat, poultry, fruits, vegetables, fats, and oils, sugar, etc.), total energy consumed (kcal), and macronutrient intake from the US Department of Agriculture (USDA) Economic Research Service (ERS), USDA Department Circular 241 (1929), US Department of Commerce (USDC) Historical Statistics of the US 1789–1945 (1949), USDA Food Consumption 1909–1952 (1953), USDA The National Food Situation NFS-74 (1956), and the National Bureau of Economic Research's *The Changing Body* (2011) (31–36). The resources with their respective data availability are summarized in Table 1. Additional sources were used when estimating availability of processed and ultra-processed foods.

**Table 1:** Data sources and time periods.

**Source**	**Time periods**	**Available food data**
USDA ERS (not loss-adjusted)	1909–2017	All food groups (red meat, poultry, fish and shellfish, dairy-fluid and cream, dairy products, eggs, fats and oils-added^*^, fresh fruits^†^, fresh vegetables^†^, grains, sugars and sweeteners) and all macronutrient data presented in tables (carbohydrates, fats, proteins, and total calories)
USDA Department Circular 241 (1929)	1900–1928	Red meat and lard only
US Department of Commerce Historical Statistics of the US 1789–1945 (1949)	1899–1945	Red meat, poultry, dairy-fluid and cream, dairy products, eggs, fats and oils (added), fresh fruits, fresh vegetables, grains, sugars, and sweeteners
USDA Food Consumption 1909–1952 (1953)	1909–1952	Red meat, poultry, fish and shellfish, dairy-fluid and cream, dairy products, eggs, fats and oils (added), fresh fruits, fresh vegetables, grains, sugars, and sweeteners
USDA The National Food Situation NFS-74 (1956)	1935–1939, 1947–1949, 1952–1955	Red meat, poultry, fish and shellfish, dairy-fluid and cream, dairy products, eggs, fats and oils (added), fresh fruits, fresh vegetables, grains
NBER's *The Changing Body* (2011)	1800–1920 (by decade years only)	Red meat, poultry, dairy-fluid and cream, dairy products, eggs, fresh fruits, grains
USDA ERS (loss-adjusted)	1970–2017	All food groups (red meat, poultry, fish and shellfish, dairy-fluid and cream, dairy products, eggs, fats and oils-added^*^, fresh fruits, fresh vegetables, grains, sugars, and sweeteners)

* *For USDA ERS Data, Due to the discontinuation of the Census Bureau's Current Industrial Reports (CIR) in 2011, data for added fats & oils (except butter), durum flour, and candy & other confectionery products are no longer available. Because of this data limitation, certain summary estimates—such as per capita daily amounts of calories, food pattern equivalents (or servings), and food loss at the retail and consumer levels in the United States—cannot be calculated beyond 2010*.

† *Fresh fruit and vegetable data were only available from 1970 and on*.

For years with multiple data sources, an average was calculated to estimate food availability. Semi-centennial averages are summarized in Table 2. All numbers are average food availability per capita per year, unless otherwise stated.

**Table 2:** Change in food availability from 1800 to 2000, bicentennially (%).

**Year**	**Red meat (lbs.)**	**Poultry (lbs.)**	**Fish and shellfish (lbs.)**	**Dairy (fluid and cream) (lbs.)**	**Dairy products (lbs.)**	**Eggs (lbs.)**	**Fats and oils (added) (lbs.)**	**Fresh fruits (lbs.)**	**Fresh vegetables (lbs.)**	**Grains (lbs.)**	**Caloric sweeteners (lbs.)**
1800	197.63	15.07	ND	333.26	333.26	9.47	ND	80.00	ND	ND	ND
1850	189.42	15.01	ND	329.25	329.25	9.40	ND	63.60	ND	ND	ND
% change	−4.15%	−0.40%	N/A	−1.20%	−1.20%	−0.66%	N/A	−20.50%	N/A	N/A	N/A
1850	189.42	15.01	ND	329.25	329.25	9.40	ND	63.60	ND	ND	ND
1900	153.57	22.11	ND	458.01	458.01	33.36	ND	219.60	ND	ND	65.20
% change	−18.93%	47.30%	N/A	39.11%	39.11%	254.76%	N/A	245.28%	N/A	N/A	N/A
1900	153.57	22.11	ND	458.01	458.01	33.36	ND	219.60	ND	ND	65.20
1950	135.75	24.12	11.70	316.70	411.32	48.63	53.09	103.70	123.10	166.50	110.84
% change	−11.60%	9.10%	N/A	−30.85%	−10.19%	45.77%	N/A	−52.78%	N/A	N/A	70.00%
1950	135.75	24.12	11.70	316.70	411.32	48.63	53.09	103.70	123.10	166.50	110.84
2000	120.15	77.40	15.20	210.51	382.83	32.24	84.23	128.69	185.90	199.46	148.88
% change	−11.49%	220.85%	29.88%	−33.53%	−6.93%	−33.70%	58.67%	24.09%	51.02%	19.80%	34.32%
1800	197.63	15.07	ND	333.26	333.26	9.47	ND	80.00	ND	ND	ND
2000	120.15	77.40	15.20	210.51	382.83	32.24	84.23	128.69	185.90	199.46	148.88
% change	−39.20%	413.60%	N/A	−36.83%	14.88%	240.59%	N/A	60.86%	N/A	N/A	N/A

*ND, No data available*.

*N/A, Not applicable as the percent change couldn't be calculate it at least one data point was missing*.

*Sources: Numbers were averages calculated from published data that were not adjusted for food loss: USDA ERS, USDA Department Circular 241 (1929), USDC Historical Statistics of the US 1789–1945 (1949), USDA Food Consumption 1909–1952 (1953), USDA The National Food Situation NFS-74 (1956), and The Changing Body (2011)*.

Food availability and consumption estimates before 1909 relied primarily on historical sources (i.e., books, articles, personal food diaries) since no systematic data are available; there was very little quantitative data on red meat, poultry, dairy, and eggs (37–45).

For estimates from 1909 to the present, the USDA ERS was our primary source, with data gathered directly from producers and distributors, tracking annual commodity production to end products. Per capita food availability estimates were first published in 1941 to assess WWII resources and a historical series for 1909–1940 was retrospectively created. Since 1941, yearly per capita food availability estimates were published.

Per the USDA ERS, the *Total annual food supply of a commodity* = *Available commodity supply (production* + *imports* + *beginning stocks) - Measurable nonfood use (farm inputs* + *exports* + *ending stocks, etc.)* and *Per capita availability* = *Total annual food supply of a commodity / U.S. population for that year (from the US Census Bureau)* (31).

Classification of food processing is based on NOVA criteria (46, 47). We used per capita food availability estimates interchangeably with per capita food consumption, as the USDA used these data as proxies for actual consumption at the national level.

These data sources provide per capita estimates on consumption of energy, nutrients, and non-nutrient food components from foods and beverages from 1909 to 2019. These nutrient intakes do not include dietary supplements or medications. We graphed data trends over time and calculated percent changes.

We also included USDA loss-adjusted food availability data from 1970 to 2019, calculated by estimating food loss at primary, retail, and consumer levels. Primary losses occurred from farm to retail weight. Retail losses occurred at supermarkets, convenience stores, and small grocery stores. Consumer losses included food discarded at home or at restaurants, including expired foods, non-edible portions (e.g., apple cores), loss from cooking and plate waste (31). Loss-adjusted food availability data from 1970 to 2019 were analyzed separately.

### Data Limitations

Data from the USDA, USDC, and other sources used different methodologies which varied over time. For example, some values gathered from different time periods were reported in different units, and conversions are not easily applied as food and beverage weight and volume measurements differ greatly. We reviewed all identified USDA and USDC, US Government Printing Offices and other federal websites, and historical sources from the nineteenth and early twentieth centuries. Despite the historical sources, we lacked data from 1800 to 1908 for fish and shellfish, added fats and oils, grains, and vegetables. We also lacked data on sugars and sweeteners from 1800 to 1874. USDA availability data from 2019 were also not available at the time of analysis for fresh fruits and vegetables and for fish and shellfish.

USDA ERS availability data from 2010 and onward for durum flour (embedded in total wheat flour) and added fats and oils were missing due to the termination of select Current Industrial Reports (CIR) by the Census Bureau. Data for rice are unavailable after 2010 due to a large unexplained decline in the implied total domestic and residual use (unreported losses in the milling, transporting, and marketing of rice) estimates. Thus, per capita daily amounts of calories and food pattern equivalents could not be calculated beyond 2010 for the added fats & oils group, as well as the summary estimates or totals across all food groups (31).

USDA ERS 1909–1941 food availability data used retrospective estimates without referenced sources. Data were likely more accurate for products primarily imported and taxed like sugar, or those entering the US during specific times: e.g., industrial seed (vegetable) oils and margarines after 1910 and high fructose corn syrup (HFCS) in the 1970s. Ready-to-eat cereal data were not available from 1989 to 2012. The USDA ERS acknowledged inaccuracies due to “incomplete reporting, inaccurate conversion factors, and inappropriate estimation techniques,” as well as information on retailers' and wholesalers' inventories. Food availability data do not include information on processing before sale, where the foods were sold, how they were prepared and eaten, or consumer profiles (31).

Changing inclusion criteria for food availability data limited some comparisons across different periods. The USDA included the military in average food availability estimates until 1941 but excluded the military afterwards; this accounted for the dramatic decrease in certain foods from 1941–1945, together with civilian quotas (31).

The 1909–1941 USDA ERS estimates did not accurately track foods consumed before transport, underrepresenting local produce and meats from farms at a time when many Americans lived on or near farms. For example, in 1920, 30.2% of Americans lived on farms and many others lived nearby (48). Consumption of offal (organ) meats, marrow, feet, snouts, “spam,” and bloods was rarely tracked and varied over time and socioeconomic groups (49). Farm to retail and consumer-level food loss estimates were imprecise (50). The USDA ERS data estimated commercial vegetables and home-grown vegetables from the early 1970s and the present, although home-garden data are unreliable (51, 52).

USDA ERS loss-adjusted food availability data were published after 1970 but were unreliable. Consumer and household surveys suffered from recall bias and variations in food loss over time due to increased fat trimming from meats or greater shelf-life due to preservatives were not accurately assessed (31, 50). We only included Nationwide Food Consumption Surveys (NFCS) and National Health and Nutrition Examination Survey (NHANES) data for non-nutritive sweeteners. The surveys only started reporting data in 1965 (NFCS) and 1999 (NHANES) and were limited by biases, inaccurate recall, mis-estimates of energy consumption, and internal inconsistencies (53).

## Results

### Total Caloric and Macronutrient Intake (Per Capita Per Day)

**Figure 1:**
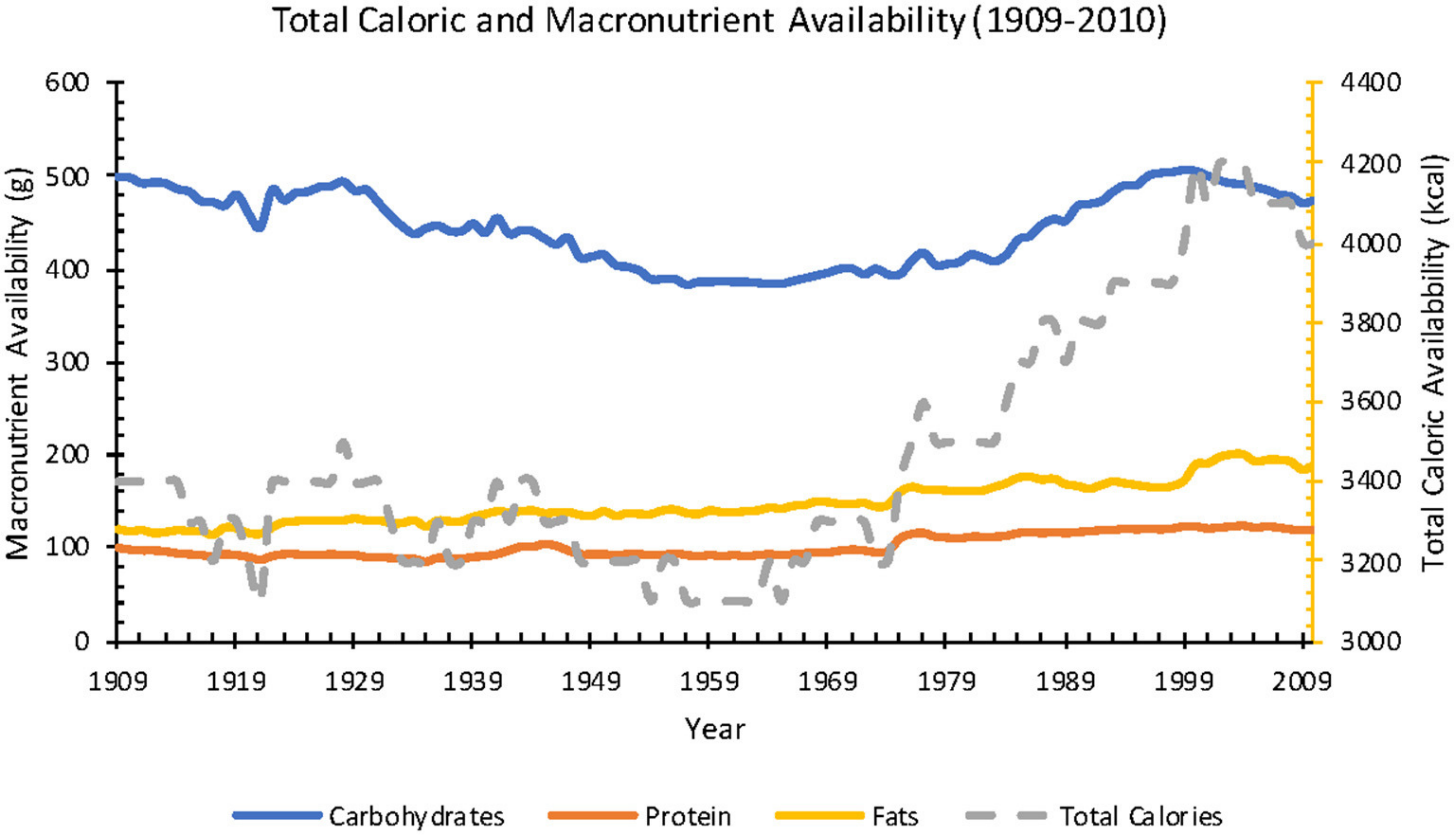
Annual total caloric and macronutrient availability per capita from 1909 to 2010 (Source: *USDA ERS*).

**Figure 2:**
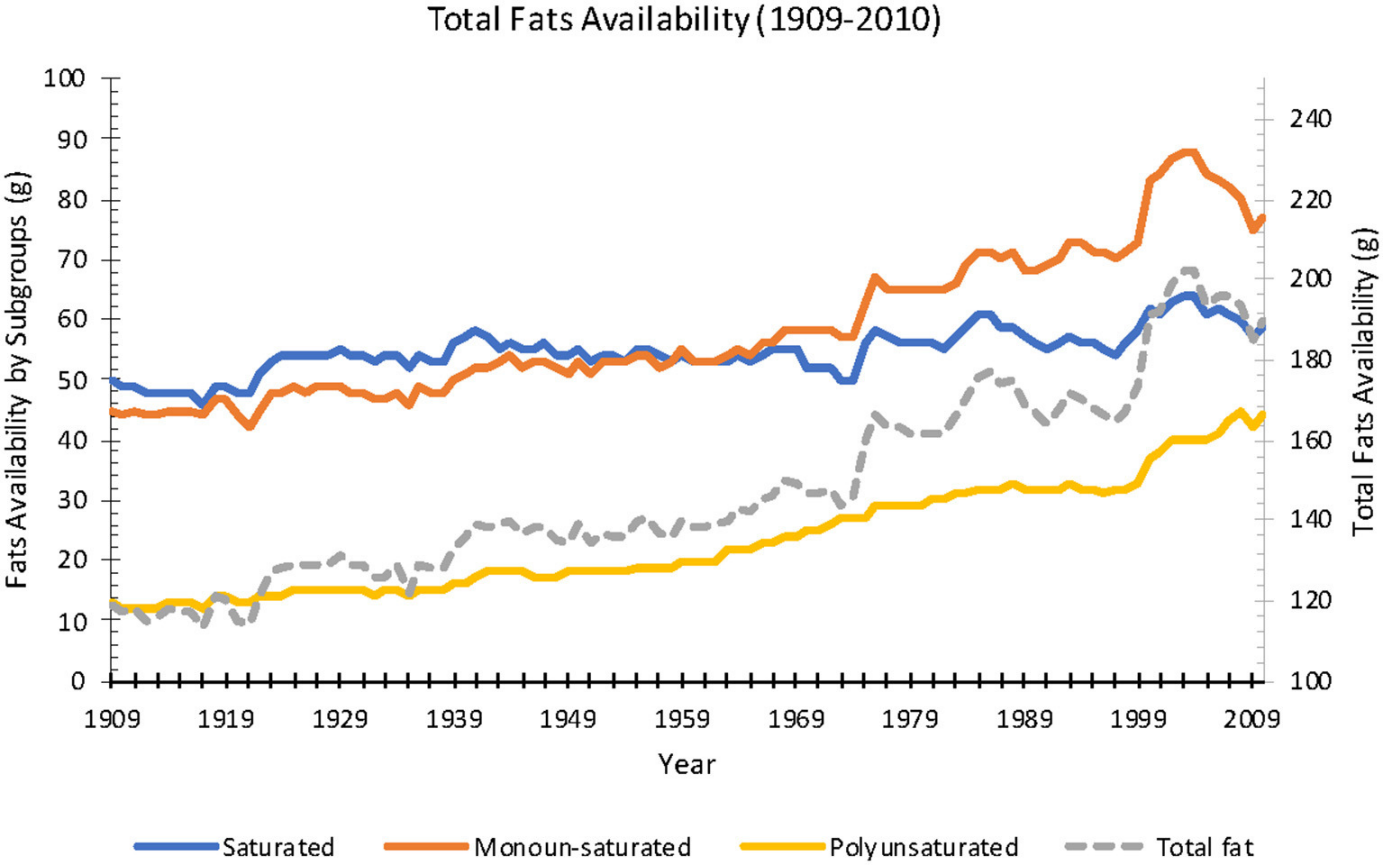
Annual fats availability per capita from 1909 to 2010 (Source: *USDA ERS*).

Available daily calories averaged 3,400 kcal in 1909 and increased 18% over the century to 4,000 kcal in 2010. In the same time period, carbohydrate availability decreased 5% (499–474 g), protein availability increased 173% (101–120 g), and fat availability increased 60% (119–190 g) (Figure 1). Saturated fat increased 18% (50–59 g), monounsaturated fat increased 71% (45–77 g), and polyunsaturated fat increased 238% (13–44 g) (Figure 2).

**Figure 3:**
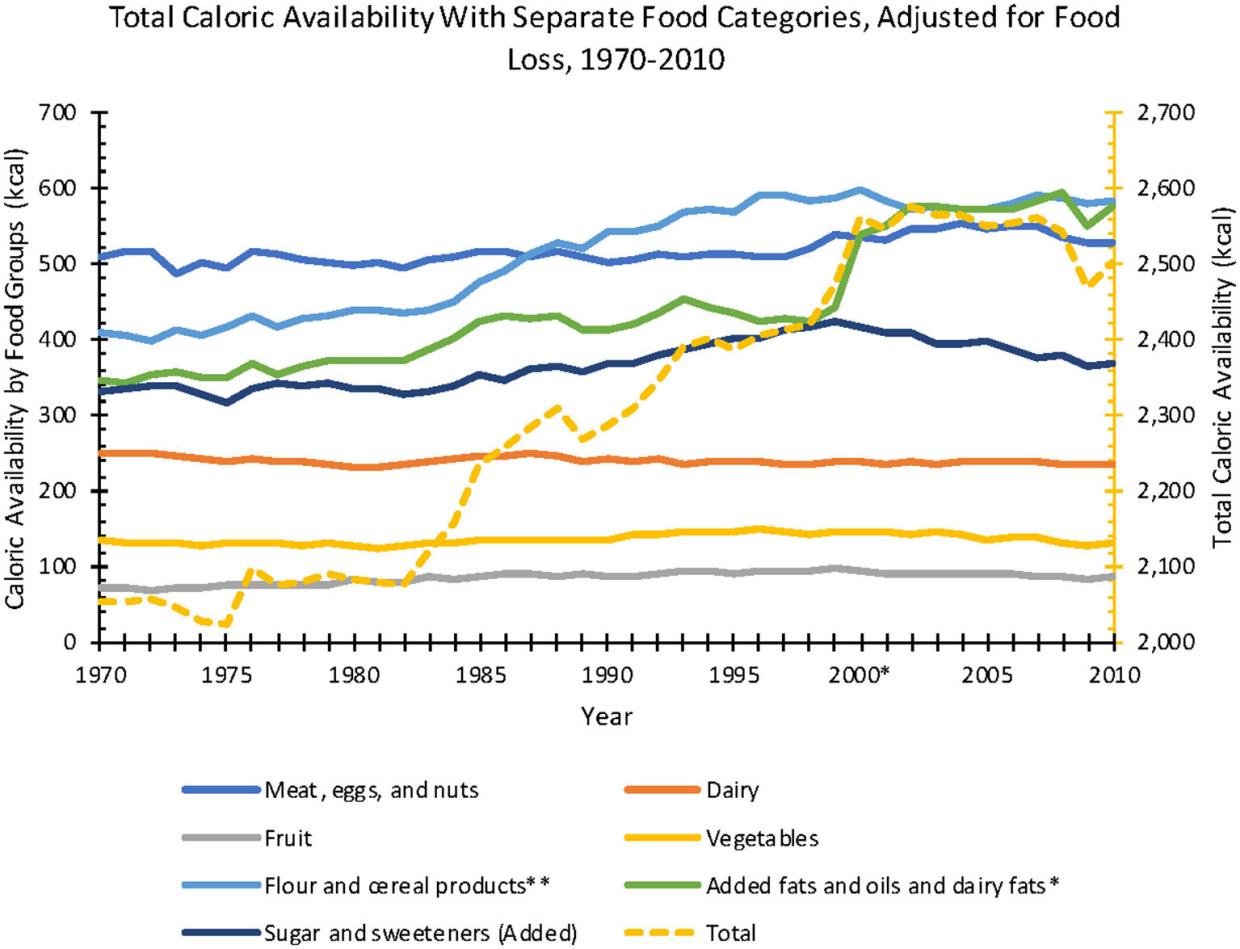
Annual total caloric availability per capita, adjusted for food loss, from 1970 to 2010 (Source: *USDA ERS*).

From 1970 to 2010, USDA reported that total caloric availability data adjusted for loss increased 22% (2,054–2,501 kcal). During this period, estimated availability adjusted for loss revealed the greatest percent increases for: added fats and oils and dairy fats (346–575 kcal; 66% increase), flour and cereals (410–581 kcal; 42% increase), fruit (71–86 kcal; increased 21%), added sugar and sweeteners (333–367 kcal; 10% increase), meat, eggs, and nuts (509–526 kcal; 3%); and losses for dairy (250–235 kcal; 6% decrease) and vegetables (135–130 kcal; 4% decrease) (Figure 3).

### Red Meat

**Figure 4:**
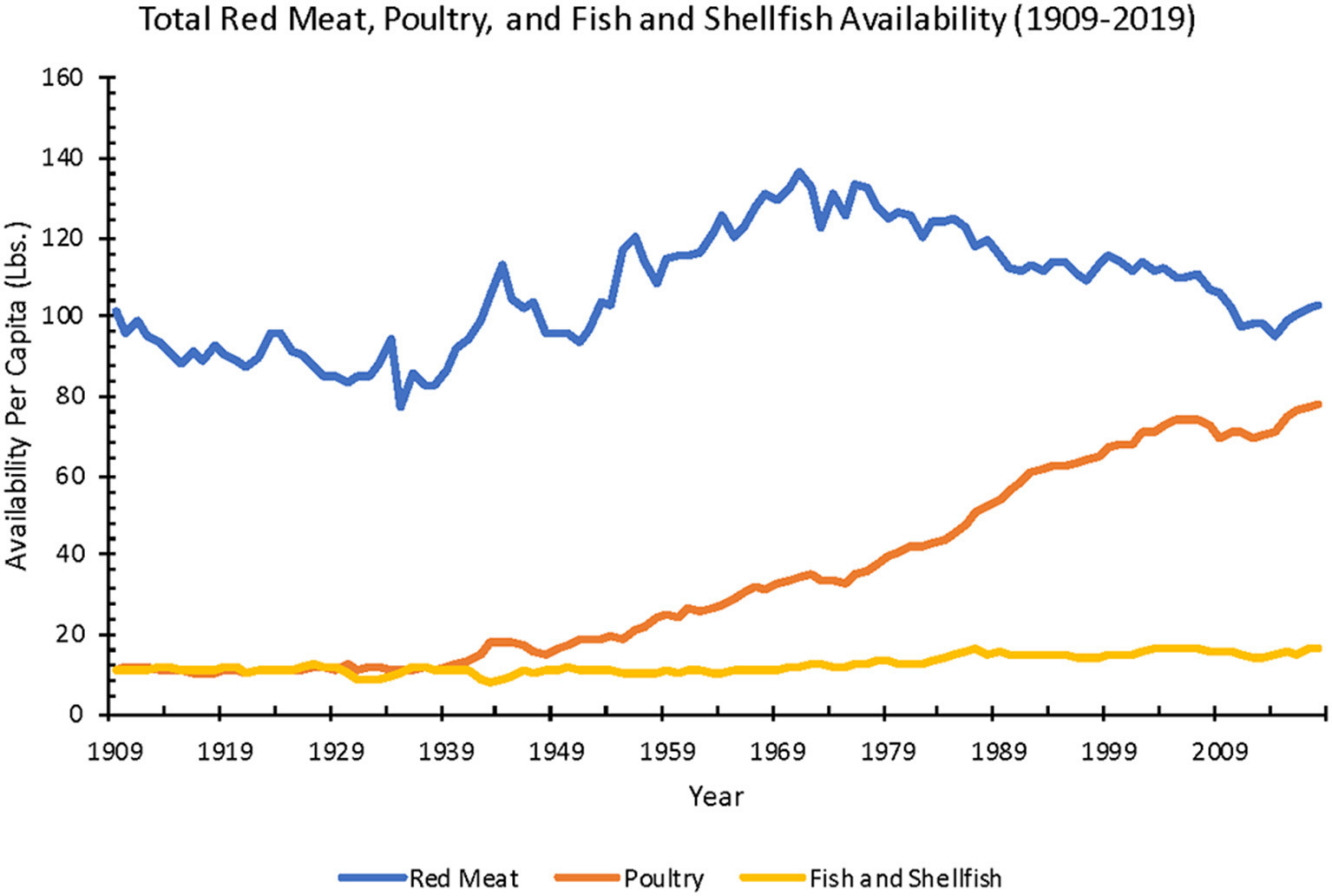
Annual red meat, poultry, and fish and shellfish availability per capita from 1909 to 2019 (Source: *USDA ERS*).

Estimated red meat availability decreased over the past two centuries. Red meat (beef, pork, lamb, veal, venison) availability per capita declined 44% from 1800 to 2019 (198–111 lbs.) (Figure 4). From 1970 to 2019, after adjusting for food loss, annual per capita red meat consumption declined 21% (96.8–76.0 lbs.).

### Poultry

Estimated chicken and turkey availability increased 550% from 1800 to 2019 (14.9–96.8 lbs.). After adjusting for food loss, from 1970 to 2019, annual per capita poultry availability increased 136% (26.4–62.4 lbs.).

### Dairy Products

Average per capita dairy product availability increased 96% from 1800 to 2019 (333–652 lbs.). Cheese availability increased 908% from 3.8 lbs. in 1909 to 38.3 lbs. in 2019. From 1970 and 2014, adjusting for food loss, total dairy products decreased by 34% (232–154 lbs.) and cheese increased 209% (8.0–24.7 lbs.).

Whole milk and cream availability decreased 56% from 1800 to 2019 (323–142 lbs.). After adjusting for food loss, between 1970 and 2017, milk and cream availability decreased 49% from 188 to 96.6 lbs.

### Eggs

Estimated egg availability per capita increased 297% from 1800 to 2019 (9.5–37.7 lbs.). After adjusting for food loss, between 1970 and 2019, egg availability decreased 5% (24.9–23.7 lbs.).

### Fresh Fruits and Vegetables

Fresh fruit availability increased 26% from 1800 to 2018 (80.0–140 lbs.). Adjusting for food loss from 1970 to 2018, available fresh fruit increased 33% (43.8–58.3 lbs.).

Fresh vegetable availability increased 33% from 1909 to 2018 (144–191 lbs.). Adjusting for food loss between 1970 and 2018, fresh vegetables per capita increased 8% (85.9–92.8 lbs.). Potatoes were most the most consumed vegetable. From 1970 to 2019, fresh potato availability decreased 45% (61.8–34.2 lbs.). From 1970 to 2018, fresh potato availability adjusted for loss declined 46% (41.9–22.8 lbs.).

### Fish and Shellfish

Total fish and shellfish availability increased 46% from 1909 to 2018 (11.0–16.1 lbs.). Overall, from 1909 to 2018, fresh fish and shellfish increased 186% (4.3–12.3 lbs.), canned fish and shellfish stayed at an average of 3.1 lbs., and cured fish decreased 93% (4.0–0.3 lbs.). After adjusting for food loss, from 1970 to 2018, total fish and shellfish per capita availability increased 29% (7.5–9.7 lbs.), fresh fish and shellfish increased 76% (3.8–6.7 lbs.), canned fish and shellfish stayed at an average of 3.0 lbs., and cured fish stayed the same at 0.3 lbs.

### Added Fats and Oils

Estimated total added fat and oil availability increased 118% from 1909 to 2010 (38.5–83.8 lbs.), with a striking decline in animal fat and increase in industrial seed oil and vegetable shortening from 1909 to 1970.

**Figure 5:**
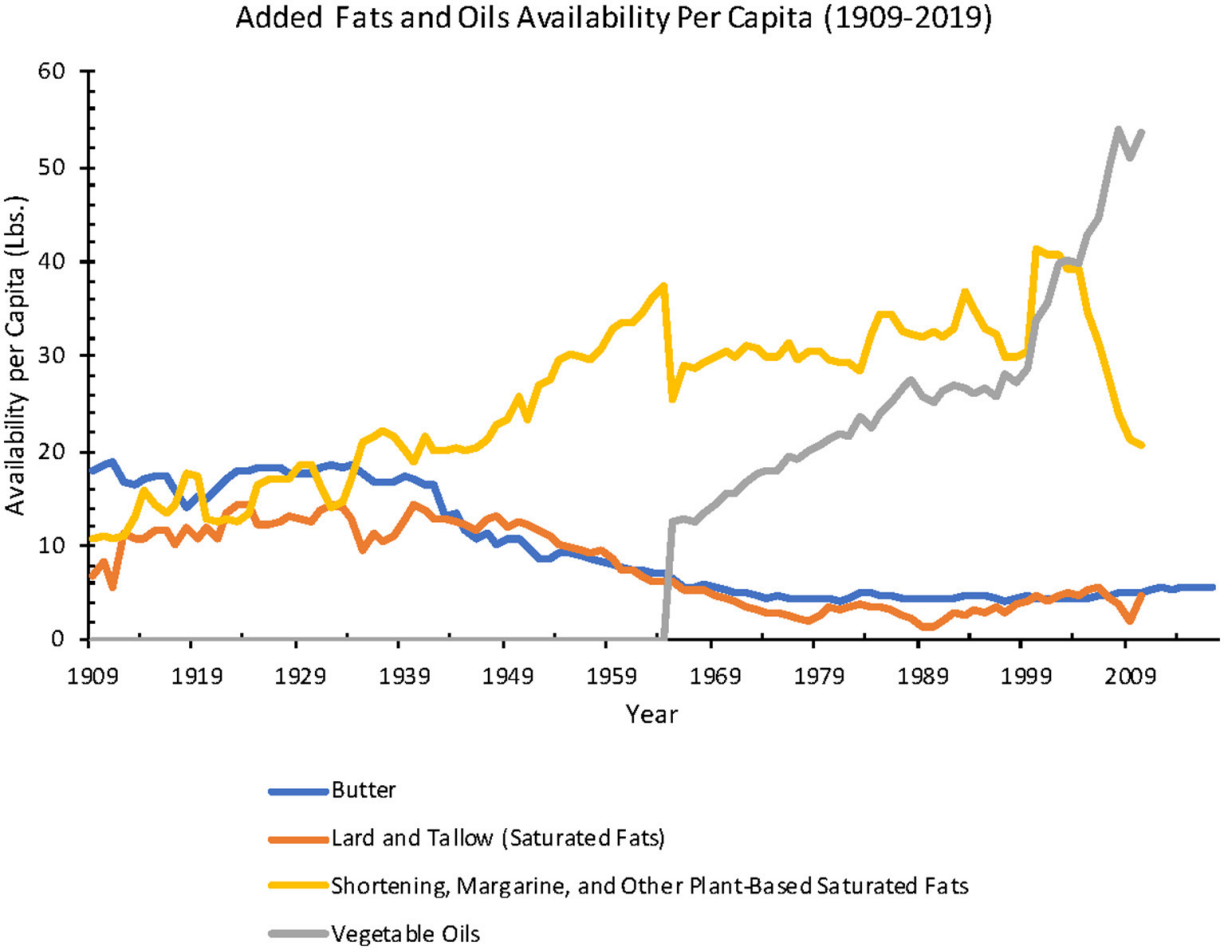
Annual added fats and oils availability per capita from 1909 to 2019 (Source: *USDA ERS*).

From 1909 to 2010, total availability per capita for animal-based fats (including butter, lard, edible tallow) decreased 58% (21.2–8.8 lbs.) and vegetable-based fats and oils (margarine, shortening, salad, and cooking oils (included only after 1965), and edible fats and oils found in confectionery products and non-dairy creamers) increased 159% (31.7–82.2 lbs.) (Figure 5).

**Figure 6:**
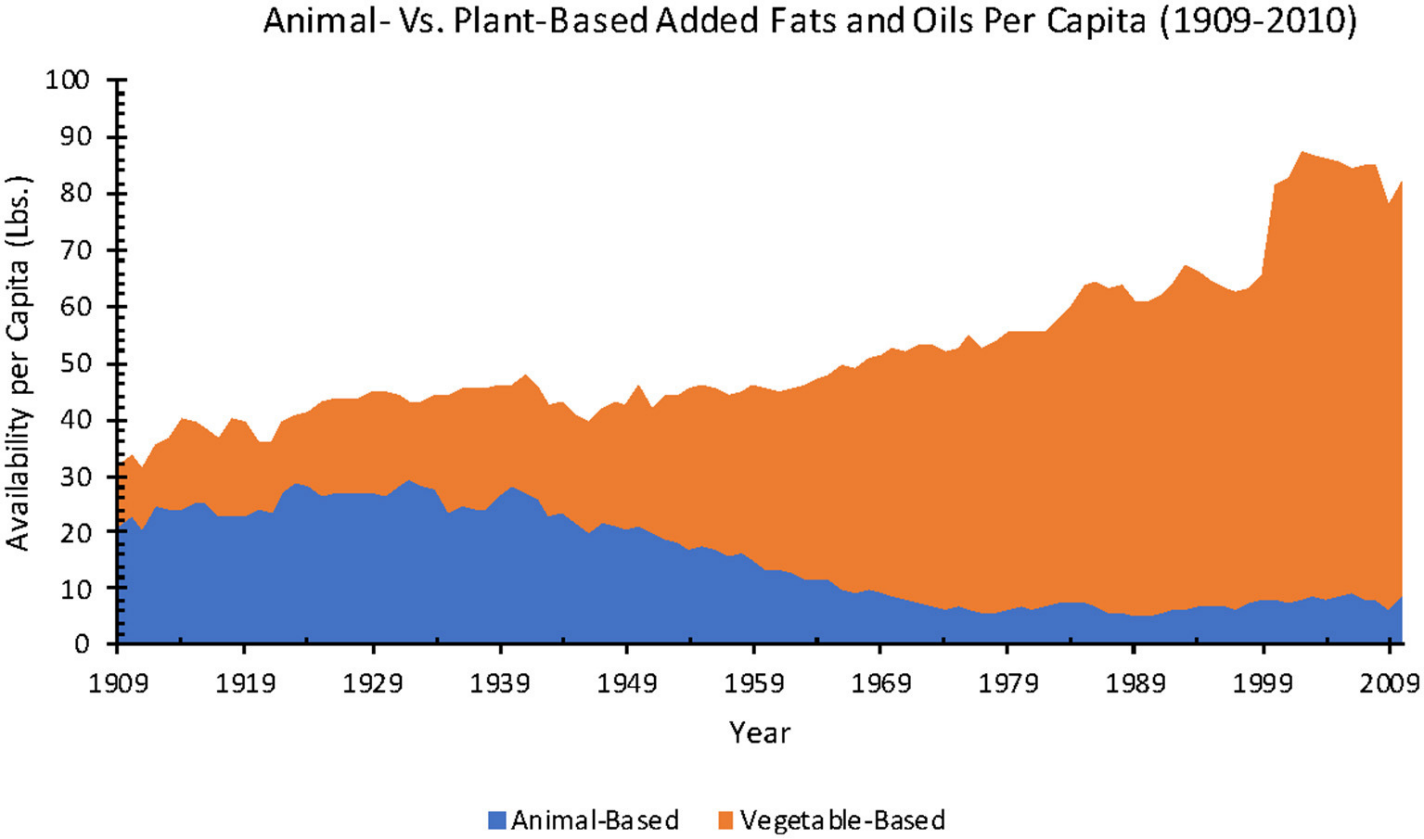
Annual animal-based versus plant-based added fats and oils availability per capita from 1909 to 2010 (Source: *USDA ERS*).

When added fats and oils were further analyzed as separate categories from 1909 to 2017, butter availability decreased 68% (17.9–5.7 lbs.), lard decreased 78% (6.9–1.5 lbs.), margarine 192% increased (1.2–3.5 lbs.), and shortening (hydrogenated oils for cooking and baking) availability increased 91% (8.0–15.3 lbs.). Salad and cooking oil availability increased 329% from 12.5 lbs. in 1965 to 53.6 lbs. in 2010 (Figure 6).

When adjusted for food loss, between 1970 and 2010, total added fat and oil availability per capita increased 61% (32.1–51.7 lbs.). In the same time frame, for separate subcategories, butter availability stayed at an average of 3.3 lbs., lard decreased 67% (1.5–0.5 lbs.), margarine decreased 68% (6.6–2.1 lbs.), shortening decreased 11% (8.9–7.9 lbs.), and salad and cooking oils increased 250% (10.3–36.0 lbs.).

**Figure 7:**
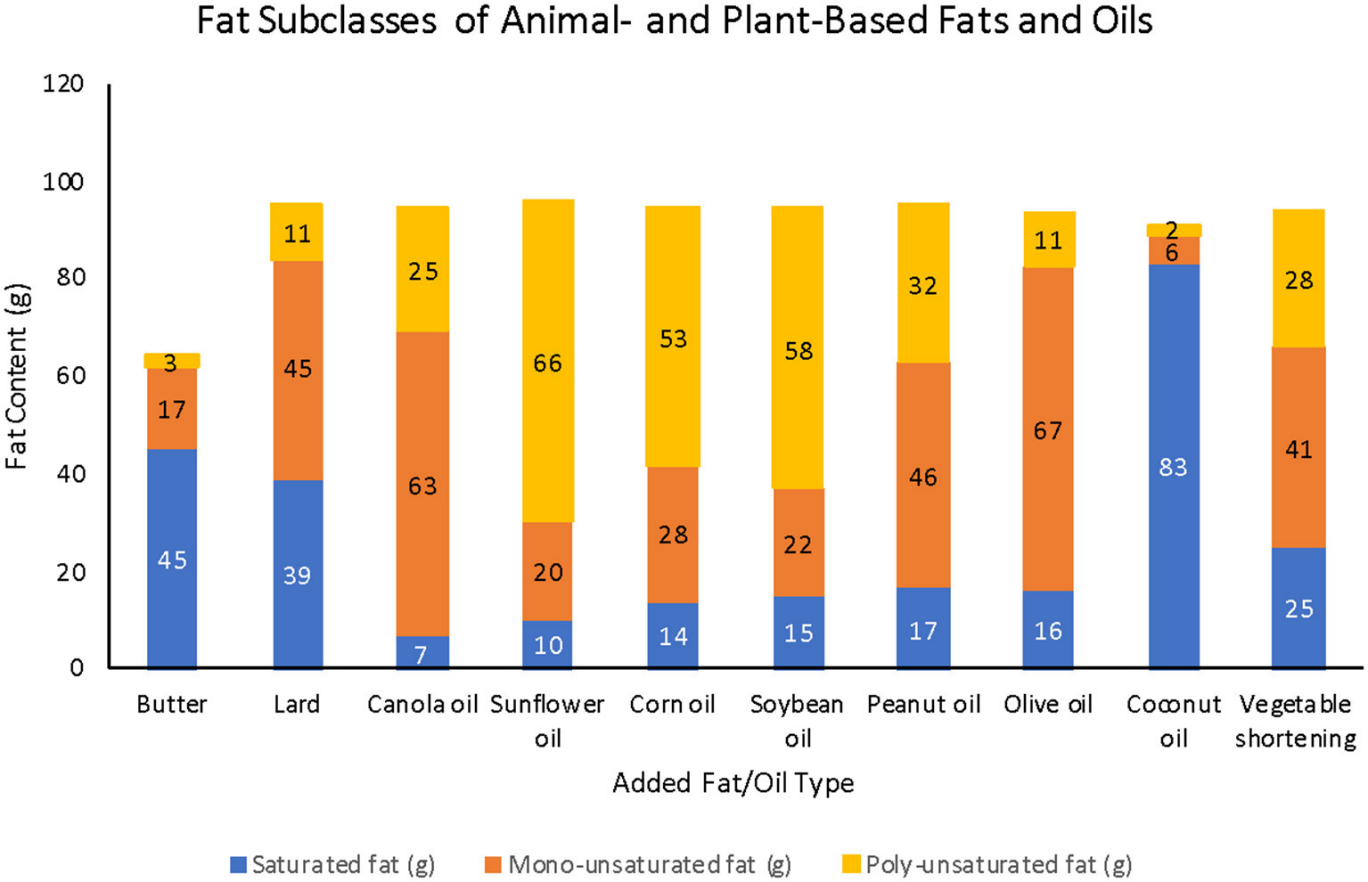
Fat subclasses of various animal- and plant-based fats and oils (Source: *USDA Food Central Data*).

The subclasses of fats that make up the different added fats and oils were also considered. One hundred grams of butter contains 65 g of total fat with 45.5 g saturated fatty acids (SFAs), 17 g monounsaturated fatty acids (MUFAs), and 2.5 g polyunsaturated fatty acids (PUFAs), simplified as 65 g/45.5 g/17 g/2.5 g for total fat/SFAs/MUFAs/PUFAs. Lard (100 g) contains 100 g/39 g/45 g/11 g of fat. The fat content of margarine depends on the type of vegetable oil that is used. Common vegetable oils used either in margarine or standalone include canola (100 g/6.5 g/63 g/25 g), sunflower (100 g/10 g/20 g/66 g), corn (100 g/13.5 g/28 g/53 g), soybean (100 g/ 15 g/22 g/57.5 g), or peanut (100 g/17 g/46 g/32 g). Other common vegetable oils include olive oil (100 g/16 g/ 66.5 g/11 g) and coconut oil (99 g/83 g/6 g/2 g). Finally, vegetable shortening include 100 g/25 g/41 g/28 g of fat (Figure 7) (54).

### Grains

From 1909 to 2010, estimated grain (wheat flour, rye flour, rice, corn, oat, and barley products) availability per capita decreased 28% (268–194 lbs.). From 2011 to 2019, rice estimates were not reported by the USDA ERS due to the termination of select Current Industrial Reports (CIR) by the Census Bureau. By excluding rice, the total grain availability from 2011 to 2019 stayed at an average of 173 lbs. After adjusting for food loss from 1970 to 2010, total grain availability (including rice) per capita increased 41% (94.8–134 lbs.).

Per capita availability data on select type of grains are also available from 1967 to 2019: total wheat flour (white, whole wheat, and durum flour) increased 16% (113–131 lbs.), rye flour decreased 58% (1.2–0.5 lbs.), corn products increased 179% (13.1–36.5 lbs.), oat products stayed the same at 4.8 lbs., and barley products decreased 42% (1.2–0.7 lbs.). Rice availability increased 158% from 1967 to 2010 (7.9–12.5 lbs.); data after 2010 are not available due to the termination of CIR reports. Adjusted for food loss from 1970 to 2019, total wheat flour increased 18% (78.0–92.3 lbs.), rye flour decreased 60% (0.85–0.34 lbs.), corn products increased 229% (7.8–25.7 lbs.), oat products stayed the same around 2.9 lbs., and barley products decreased 29% (0.63–0.45 lbs.). Loss-adjusted rice availability from 1970 to 2010 increased 161% (4.6–12.0 lbs.).

### Beverages

Beverage availability fluctuated over time from 1910 to 2015, depending on the type of beverage.

#### Coffee, Tea, and Chocolate Liquor

**Figure 8:**
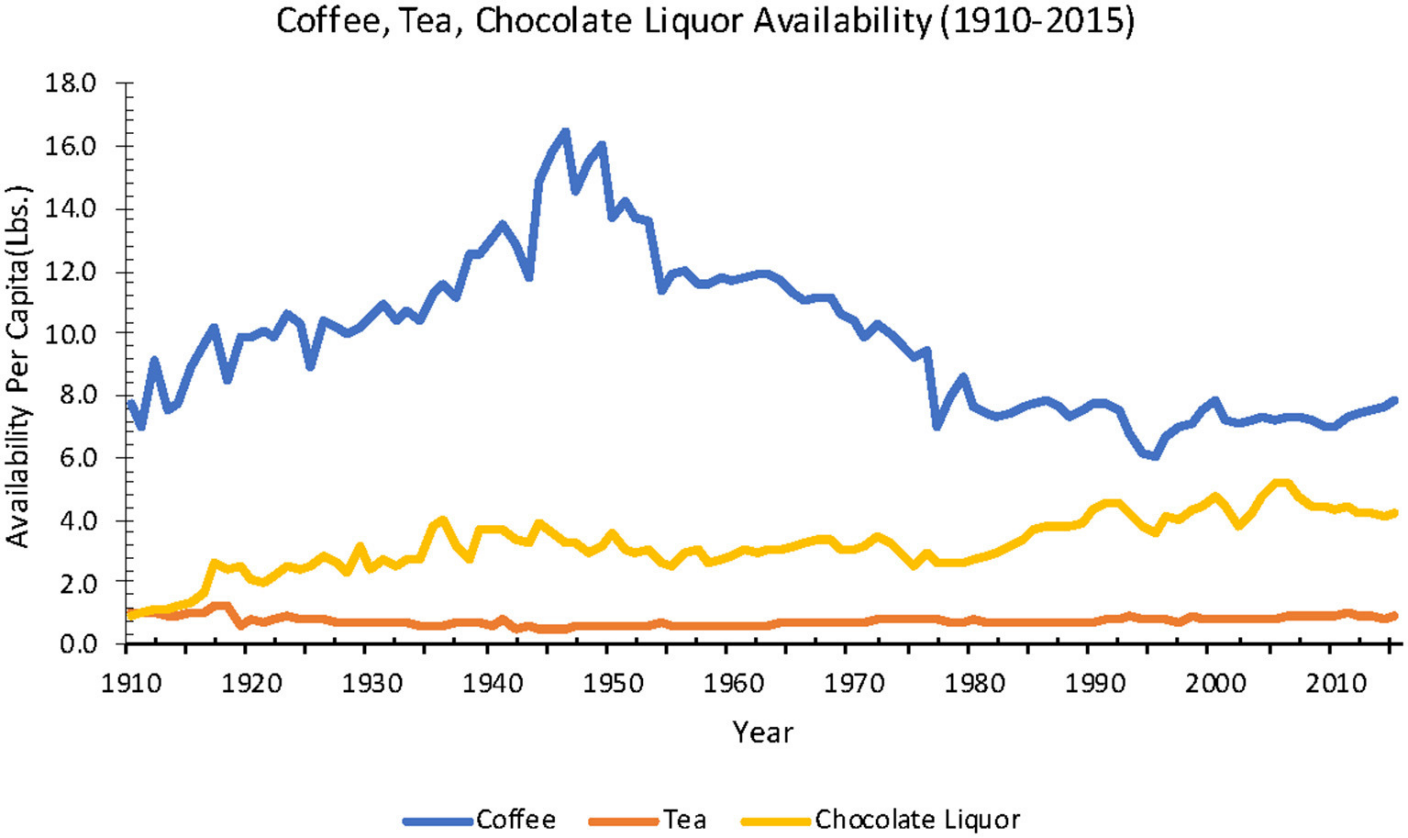
Beverage (Coffee, Tea, Fruit Juices) availability from 1909 to 2017 in the United States of America (Source: *USDA ERS*).

Coffee availability in retail weight, including both regular and instant coffee (available since 1951) from 1910 to 2015 had an average of 7.8 lbs. per capita per year. From 1910 to 2015, dry leaf tea availability averaged 1.0 lb., while chocolate liquor, or the remaining substance after cocoa beans have been roasted and dehulled (ground or bitter chocolate), availability increased 378% (0.9–4.3 lbs.) (Figure 8). Loss-adjusted data were not available for coffee, tea, and chocolate liquor.

#### Alcohol

Per capita consumption for all alcoholic beverages (including beer, wine, and spirits) increased 14% from 1850 to 2019 (2.1–2.4 gal) (55).

**Figure 9:**
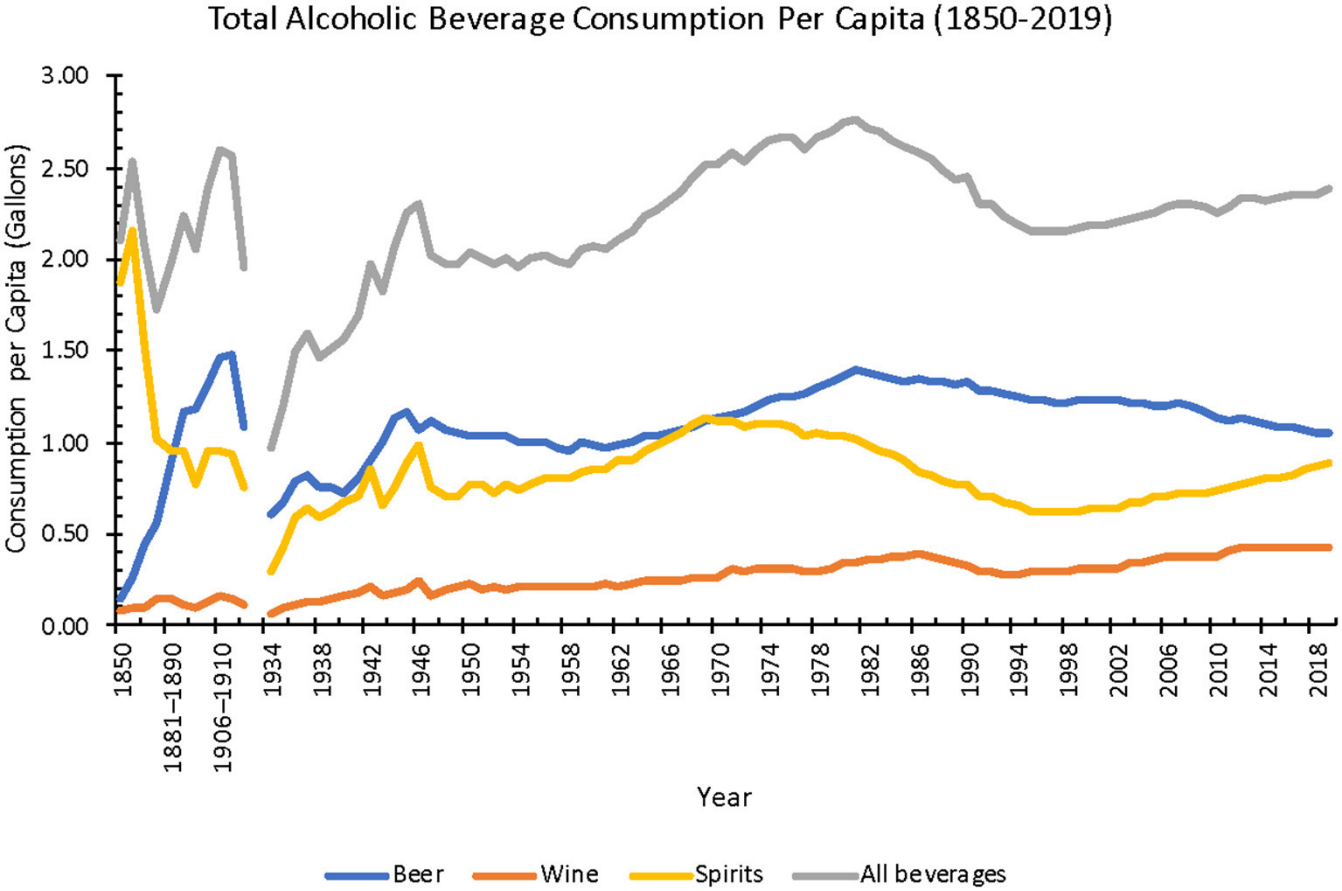
Annual total alcoholic beverage consumption per capita from 1850 to 2019 (Source: *NIAAA*). The gap in data is due to Prohibition from 1920 to 1933.

Individual alcoholic beverages exhibited varied trends, before prohibition from 1850 to 1919 and after it ended in 1933. From 1850 to 1919, beer consumption increased 900% (0.1–1.0 gal), whereas spirits consumption decreased 58% (1.9–0.8 gal). Wine consumption at this time stayed the same at an average of 0.1 gal. After prohibition ended in 1933, from 1934 to 2019, beer consumption increased 67% (0.6–1.0 gal), spirits increased 200% (0.3–0.9 gal), wine increased 300% (0.1–0.4 gal). Overall, from 1850 to 2019, beer consumption increased 650%, spirits consumption decreased 53%, and wine consumption increased 438% (Figure 9) (55).

#### Sugar-Sweetened Beverages

Sugar-sweetened beverages (SSB) include any beverage with added sugar, including but not limited to non-diet soft drinks/sodas, flavored juice drinks, sports drinks, sweetened tea, coffee drinks, energy drinks, and electrolyte replacement drinks (56). From 1950 to 2000, SSB consumption capita increased 356% (10.8–49.3 gal) (57).

Additional data from the NFCS and NHANES estimated a 306% increase from 1965 to 2002 in SSB caloric consumption per capita per day (50–203 kcal) among the general population. In the same time frame, the percentage of people reported to have had consumed SSB increased from 29.2 to 59.6%. And, among SSB consumers only, the average SSB calories consumed per day increased 86% (173–321 kcal) (58).

However, from 1999 to 2010, a decrease in SSB consumption has been observed in both the youth (2–19 years old) and adults (≥20 years old). Among the youth, total daily caloric intake from SSBs decreased 30.5% (223–155 kcal) from the 1999–2000 to the 2009–2010 survey period, respectively. Similarly, among adults over the same time period, daily caloric intake from SSBs decreased 20.9% (196–151 kcal) (59).

### Sweeteners

#### Caloric Sweeteners

Estimated total caloric sweetener (cane and beet sugar, corn sweeteners, edible syrups, and honey) availability increased 206% from 1875 to 2019 (40.3–123.2 lbs.). When adjusted for food loss from 1970 to 2019, the estimated total caloric sweetener availability increased 4% (70.2–72.7 lbs.).

**Figure 10:**
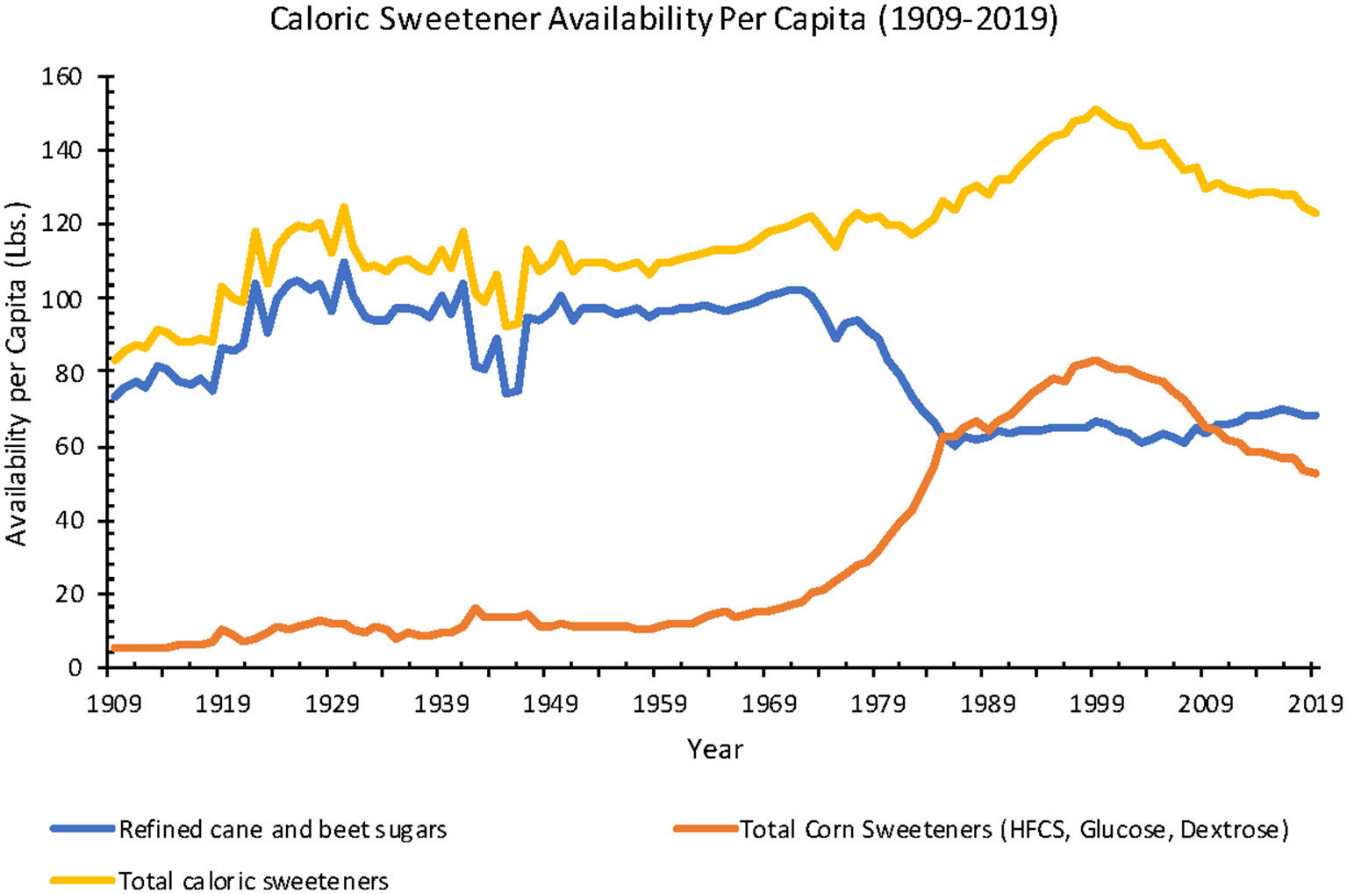
Annual caloric sweetener availability per capita from 1909 to 2010 in the United States of America (Source: *USDA ERS)*.

High-fructose corn syrup (HFCS) entered the American market in the late 1960's, with total per capita availability at around 1 lb. in 1967 before increasing ~3,570%−36.7 lbs. in 2019. In comparison, from 1967 to 2019, refined beet and cane sugar availability decreased 31% (98.5–68.4 lbs.), glucose increased 33% (9.9–13.2 lbs.), dextrose decreased 33% (4.3–2.9 lbs.), edible syrup increased 60% (0.5–0.8 lbs.), and honey increased 44% (0.9–1.3 lbs.). When adjusted for food loss from 1970 to 2019, the estimated HFCS availability increased 710% (0.3–21.6 lbs.), refined beet and cane sugar availability decreased 33% (59.8–40.2 lbs.), glucose availability increased 22% (6.3–7.7 lbs.), dextrose availability decreased 37% (2.7–1.7 lbs.), edible syrups availability increased 50% (0.4–0.6 lbs.), and honey availability increased 25% (0.8–1.0 lbs.) (Figure 10).

#### Non-nutritive Sweeteners

In estimates gathered from the NFCS and NHANES, total non-nutritive sweetener consumption per capita increased 1,227% from 1965 to 2004 (11–146 g). In the same time frame, the percentage of the US population who reported to have had consumed foods and beverages containing non-nutritive sweetener increased from 3.3 to 15.1%. When considering the average consumption of non-nutritive sweeteners among only those who reported to consume them, the average consumption increased 118% (304–663 g) per consumer (60).

The Nielsen Homescan Household Panel estimated that from 2002 to 2018, the total number of households purchasing only non-nutritive sweetener-containing products increased slightly (65.7–67.2%), and the proportion of households that purchased products containing both caloric and non-nutritive sweeteners also increased (46.8–74.1%). During this period, saccharin consumption decreased 50% (0.6–0.3 g), aspartame decreased 16% (94.7–80.0 g), reb-A increased 760% (0.0–7.6 g), sucralose increased 221% (15.4–49.4 g), and all other non-nutritive sweeteners increased 128% (40.3–91.9 g) (61).

### Processed and Ultra Processed Foods

The 2014 consensus classification of unprocessed/minimally processed, processed ingredients, ready-to-eat food products, and ultra-processed foods cannot be applied systematically to earlier periods (51). There was a dramatic increase in processed and ultra-processed food consumption in the United States over the twentieth century, including ingredients (e.g., seed oils, white flour, rice, sugars, and syrups), food products (e.g., canned or bottled vegetables and legumes in brine; peeled or sliced fruits in syrup; tinned fish in oil; salted nuts; ham, bacon, smoked fish, cheese), and ultra-processed products (e.g., chips, pretzels, ice cream, hot dogs and hamburgers, chicken fingers, cereals, cakes, energy bars, pizza, sodas and sports drinks, fruit yogurts) (37).

#### Processed Fruits

Processed fruit availability from 1970 to 2018 decreased 26% (137–101 lbs.), frozen fruit increased 26% (3.9–4.9 lbs.), canned fruit decreased 54% (26.2–12.0 lbs.), fruit juice decreased 17% (96.7–80.0 lbs.), and dried fruits decreased 70% (10.0–3.0 lbs.). During this period, adjusted for food loss, frozen fruit availability per capita increased 39% (2.3–3.2 lbs.), canned fruit decreased 57% (19.6–8.5 lbs.), fruit juice stayed the same at 40.1 lbs., and dried fruits decreased 50% (1.9–0.8 lbs.).

#### Processed Vegetables

Processed vegetables data were only available from 1970 to 2018, and they increased 16% (182–211 lbs.). Further breaking down processed vegetables in the same time frame, frozen vegetable availability per capita increased 70% (43.7–74.2 lbs.), canned vegetable decreased 10% (101–91.1 lbs.), dehydrated vegetable stayed at an average of 13.4 lbs., and legumes increased 96% (7.0–13.7 lbs.). Adjusting for food loss from 1970 to 2018, among processed vegetables, frozen vegetable availability per capita increased 81% (16.1–29.1 lbs.), canned vegetable decreased 14% (37.4–32.3 lbs.), dehydrated vegetable stayed at an average of 6.0 lbs., and legumes increased 59% (5.9–9.4 lbs.).

Estimated processed potato availability has increased since 1970, while fresh potato availability decreased. From 1970 to 2019, total processed potato availability (farm weight) increased 42% (59.9–84.9 lbs.) Further breaking down processed potatoes, per capita frozen potato availability increased 81% (28.5–51.5 lbs.), chips increased 12% (17.4–19.4 lbs.), dehydrated potato increased 13% (12.0–13.6 lbs.), and canned potato decreased 80% (2.0–0.4 lbs.), which was the only decrease in a processed potato product.

From 1970 to 2019, loss-adjusted total processed potato availability (farm weight) increased 62% (16.4–26.6 lbs.). Further breaking down processed potatoes, loss-adjusted per capita frozen potato availability increased 101% (10.1–20.3 lbs.), chips increased 13% (3.9–4.4 lbs.), dehydrated potato increased 13% (1.5–1.7 lbs.), and canned potato decreased 78% (0.9–0.2 lbs.).

#### Processed Meat

Processed meat includes both red meat and poultry that has gone through salting, curing, fermentation, smoking, or had chemical preservatives added to it. Data from NHANES revealed that the average processed meat consumption per week per capita among adults (≥20 years old) increased 3% from the 1999–2000 survey period to the 2015–2016 time period (182–187 g). The most common processed meat consumed in the 2015–2016 survey period was luncheon meat (73.3 g/week), followed by sausage (45.5 g/week), hot dog (17.5 g/week), ham (17.5 g/week), and bacon (8.6 g/week) (62).

#### Processed Cereal

Processed grain availability was calculated from ready-to-eat (RTE) cereals that were introduced in the early twentieth century. Estimates for cereal availability increased 467% from 1935 to 1997 (0.3–14.3 lbs.). RTE cereal consumption was estimated at 0.3 lbs. per household (considered as two adults) per week in the 1935–1936 survey period, 0.5 lbs. in 1942, 0.6 lbs. in 1955, and 0.7 lbs. in the 1964–1965 survey period (63–67). Between 1967 and 1987, additional data report annual RTE cereal availability per capita increased 113% from 1.5 to 3.2 lbs. (68). Additional estimates from the USDA from 1980 to 1997 noted a 47% increase of RTE cereal from 9.7 to 14.3 lbs. (52).

Additional data from NHANES include the prevalence of RTE cereal consumption among adults and children, rather than average consumption per capita. Prevalence of RTE cereal consumption among US adults aged ≥18 years was 20% in the 2003–2004 cycle before increasing to 24% in the 2009–2019 cycle and subsequently decreasing to 19% in the 2015–2016 cycle. In the 2015–2016 cycle, RTE cereal contributed to 10% of total energy intake (214 kcal/day/capita out of 2,135 kcal/day/capita) in adults aged ≥18 years who are RTE cereal eaters. When combining both RTE cereal eaters and non-eaters as the whole population, the number was 2% (42 kcal/day/capita out of 2,102 kcal/day/capita) (69).

Among all children from 0.5 to 17 years old, prevalence of RTE cereal consumption was 41% in the 2003–2004 cycle before decreasing to 36% in the 2015–2016 cycle. In the 2015–2016 cycle, among children 0.5–17 years old who are RTE cereal eaters, RTE cereals make up 9% of total daily energy intake (161 kcal/day/capita out of 1,788 kcal/day/capita). When combining both RTE cereal eaters and non-eaters as the whole population, the number decreased to 3% (54 kcal/day/capita out of 1,807 kcal/day/capita) (70).

## Discussion

The American diet has changed radically in the past two centuries, with the most marked changes including increased consumption of processed and ultra-processed food (e.g., sugar, white flour, white rice, and industrial seed/vegetable oils) and poultry and reduced consumption of unprocessed foods (e.g., fresh fruits and vegetables) and animal fats (e.g., whole milk, butter, and lard). Changes in food availability over the past two centuries included (1) increased processed and ultra-processed foods, sugar, industrial seed oils, and poultry; and (2) decreased butter/lard/shortening, dairy (mainly whole fat), fresh fruits, fresh vegetables, and red meat (beef/pork). Ultra-processed foods were rare before 1900 but increased to more than 50% of the current American diet (46). SFA consumption remained relatively stable, as lard, butter, whole milk, and red meat decreased while margarine, shortening, and other vegetable-based saturated fats increased. Meanwhile, PUFA and MUFA consumption increased dramatically with the introduction of ultra-processed foods and industrial seed and vegetable oils.

The unprocessed elements of our nineteenth century diet–animal fats, whole fat dairy, fresh vegetables, and fresh fruits—were progressively replaced with more processed elements, including industrial seed oils, HFCS, and ready-to-eat snacks and meals. The data do not support the widely publicized “changing American diet” of increasing animal-derived SFAs over the first 60 years of the twentieth century (3, 11, 22, 71). Rather, polyunsaturated fats and partially hydrogenated fats from vegetable oils progressively replaced lard, butter, and other animal-derived fats. Across the twentieth century, rising rates of obesity, diabetes, heart disease, and cancer were associated with stable SFA consumption. Yet, large increases in sugar and refined carbohydrate consumption and more modest increases in total calories make refined carbohydrates and total calories more likely factors than SFA in NCD pathogenesis.

Data from the USDA and other sources have multiple and significant confounds. The more recent National Health and Nutrition Examination Surveys (NHANES) data we used to estimate processed and ultra-processed foods are considered the gold standard but their validity remains controversial, with major shortcomings (50, 73–77). Retrospective USDA estimates from 1909 to 1940 were inaccurate and unreliable, to an unknown degree. As one moves back in the nineteenth century, data are progressively scant and imprecise. Data on commodities such as fruits, vegetables, and grains are limited before 1940 by poor documentation of local sources. Historical accounts and records identify marked seasonal, geographic, and socioeconomic differences. Further, local consumption was extensive as most Americans lived on or near farms, but the data were not accurately measured in national estimates.

### The Changing American Diet: History and Influence

The increased consumption of red meat and SFAs as the cause of the heart disease epidemic was one foundation for Keys' Diet-Heart Hypothesis, strengthened by authoritative repetition, including McGovern's Senate Select Committee's *Dietary Goals for America* (1977), Science in the Public Interest's (1978) monograph *The Changing American Diet*, the New York Times columnist Jane Brody's (1985) *Good Food Book*, Surgeon General Koop's Report on Nutrition and Health (1988), and the World Health Organization's Diet, Nutrition, and the Prevention of Chronic Diseases (1990) (11, 71, 72, 78). However, neither the USDA nor other data supported this narrative (79). From 1800 to 2000, red meat consumption declined by 44%, fluid and cream dairy consumption declined by 48%, and egg consumption increased by 241%. From 1909 to 2010, lard consumption declined 78% and butter declined 68%, while margarine increased 192%, shortening increased 91%, and salad and cooking oils increase 329%. Americans consumed up to 70% fewer SFAs from animal sources by the end of the century, as obesity and diabetes epidemics emerged, alongside an increased incidence of NCDs such as cancer and heart disease (80).

The alleged increase in American SFA consumption in the twentieth century was considered the cause of the dramatic rise of non-communicable diseases (NCDs). Fats, especially SFAs, were considered uniquely toxic due to their caloric density or role in atherogenesis. Disorders linked to high fat/SFA diets included (1) overweight and obesity (too many calories with fat as main driver, insufficient exercise), (2) elevated cholesterol (from SFA), (3) hypertension (high salt and obesity), (4) colon and breast cancer (fat and SFA), and (5) diabetes (obesity and fats) (11). Yet, the rate of in NCDs continued to increase even after CDC guidelines encouraged Americans to reduce SFAs (81). Total SFA consumption increased slightly for total grams consumed while the percentage of all calories was stable (~13.2%). From 1909 to present day, SFA from animal sources declined significantly but SFA from partially hydrogenated vegetable oils (contained in shortening and processed/ultra-processed foods) increased greatly. By contrast, the average American consumed >10-fold more “heart-healthy” PUFAs and MUFAs, and added caloric sweeteners tripled across the twentieth century. Our findings suggest that SFAs are unlikely to drive obesity, diabetes, or other NCDs, although this belief is held by many leading public health organizations (80). The early data that led to the belief that SFAs were dangerous deserve scrutiny.

The 1961 Framingham Heart Study (FHS) initially reported that high cholesterol correlated with heart disease and dietary SFA was the nutrient most strongly related to elevated total cholesterol in short-term feeding studies (9). However, by 1961, the relationship between dietary fats, carbohydrates, and lipoproteins was more complex. The effects of short-term and long-term feeding studies often differ and nutrients such as sugar and SFAs affect lipoprotein fractions differently. SFAs raise high-density lipoproteins (HDL), which carry HDL-cholesterol, and high HDL levels have been shown to be potent predictors of heart disease risk than low-density lipoproteins (LDL) or total cholesterol (82). Additionally, diets rich in sugar and refined carbohydrates elevate triglycerides and inflammation (83, 84). Longer follow-ups with more patient-years from the FHS found that total cholesterol, after accounting for factors such as blood pressure and smoking, was only a risk factor in heart disease or total mortality for men under age 65 years; it was far less significant for women under age 50 years and insignificant for those older than 50 years old (85, 86). Further into the study, the FHS dietary data found that neither fat nor SFA consumption were related to cholesterol levels, coronary heart disease, or mortality (9). Subsequent studies, with larger and more diverse samples, failed to confirm the Seven Countries Study association of SFAs or fats with heart disease (28, 30, 87–89).

McGovern's Senate Select Committee's *Dietary Goals for America* (1977) was pivotal in definitively linking dietary SFAs as a major cause of heart disease, obesity, and cancer. Yet, three of eight senators dissented because many experts testified that neither total fat nor SFAs caused heart disease; rather, they interpreted the evidence as implicating sugar and refined carbohydrates in causing obesity, diabetes, and heart disease in animals and humans (11). A decade before the McGovern report, the future NIH and NHLBI directors found that the most common hyperlipidemia in cardiac patients primarily resulted from excess carbohydrates (25). Further, converging evidence revealed that metabolic syndrome results from refined carbohydrates in animals and humans.

US and international agencies and medical associations strongly supported a low-fat/low-SFA, high-carbohydrate diet for everyone over age 2 years, and through 2008, advocated sugar as healthy for diabetics and the general population (90). The strongest evidence implicating SFA remains in studies in which SFAs are replaced with MUFAs or PUFAs, and heart disease, and less often, overall mortality, were reduced, although some observational studies and randomized controlled trials challenge these findings (28–30, 91). These studies cannot assess the harmful effects of SFAs or how increased MUFAs and PUFAs may be beneficial and SFAs neutral, as suggested by population-based prospective studies (92, 93).

Untangling the causes of NCDs is complex, multifactorial, and controversially unresolved. The profound dietary changes were accompanied by other lifestyle and demographic changes, including (1) increased urbanization and population density, (2) reduced physical activity commuting to and at work, (3) longer commutes, (4) higher stress, (5) less sleep, (6) more machine and less human time, (7) higher rates of mental health disorders, (8) increased prescription and over-the-counter drug use, many of which increase appetite, and (9) higher salt intake. Increased obesity is a common precursor and risk factor for many NCDs (e.g., metabolic syndrome, T2D, heart disease, cancer, and gout) (94).

Public health and academic experts attribute obesity to a positive energy balance: caloric intake exceeding caloric expenditure and calorically dense fats were implicated in obesity pathogenesis (12, 13, 95–97). However, animal and human studies identify multiple exceptions to the energy balance hypothesis (e.g., overfeeding studies, populations with obese mothers and undernourished children, obesity on semi-starvation-e.g., 1,600 kcal/day diets, prospective studies showing decreased or stable weight despite increased calories) (26, 27, 98–105). Evidence supports both the roles of energy balance and refined carbohydrates-insulin mechanisms in obesity, with their relative roles likely varying based on genetics and other factors (106).

The energy balance hypothesis of obesity is supported by the 22% increase in available calories from 1970 to 2010 (Figure 1). There was a >30% increase in overweight Americans from 1976–1980 (25.4%) to 1988–1991 (33.3%), associated with an 11% decrease in percent of fat calories (41.0–36.6%), a 4% decrease in daily calories (1,854–1,785 kcal), and a 9.8-fold increase in high fructose corn syrup. During this period, Americans consuming low-calorie products rose from 19 to 76% while physical activity was stable (78). However, in the Women's Health Initiative study, three years after the intervention group consumed an average of 100 fewer calories per day and exercised more than the control group, the controls weighed 1.3 kg more, yet the energy balance predicted a difference of > 16 kg (30). Many impoverished populations underwent a dietary transition followed by rising obesity without any obesogenic environmental factors such as abundant dietary SFAs or labor-saving devices (Pima Native Americans in 1890–1920, Sioux Native Americans in 1920s, Jamaicans in 1970s, Zulus in Durbin, South Africa in 1960) (100, 101, 103, 107). This rising obesity in adults, mostly women, while their children were malnourished, refutes the energy balance hypothesis as adults reduce their basal metabolic rate rapidly with decreased caloric intake, while children only do so after losing 20–30% of body weight (108–111).

NCDs such as obesity, T2DM, heart disease, and cancer are rare in indigenous populations consuming native diets, even among elderly individuals (112–115). These populations consumed diverse diets, some very high in SFAs from animals (e.g., Inuit, Maasai, Plains Native Americans) or plants (e.g., Polynesians, Tokelauns), while many others consumed diets high in complex carbohydrates and very low in fats (e.g., Pueblo Native Americans, Japanese, and Chinese farmers) (116–120). Native, minimally processed diets included minimal sugar or refined carbohydrates; honey being a major exception in some populations such as the Hadza (121). When populations adopted Western diets and lifestyles, NCDs emerged and increased (113, 122–126). Commensurate with these dietary transitions in indigenous populations, our findings suggest that increased sugar and refined carbohydrate consumptions during the twentieth century in America may have played a larger role than total calories or physical activity, although this remains a speculation without accurate data on all variables.

### Future Direction

Understanding the pathogenic changes in American and other diets that drove the dramatic rise in NCDs remains one of the greatest challenges in public health. Given the challenges in obtaining accurate caloric estimates in national data, humility is needed to assess the diets of populations more than a century ago. Only well-defined changes (e.g., increased caloric sweeteners and PUFA and decreased SFA from lard and butter) can be identified. A more complete understanding of dietary and lifestyle factors in NCDs may emerge from an unbiased synthesis of the diverse evidentiary lines.

## Data Availability Statement

The original contributions presented in the study are included in the article. Further inquires can be directed to the corresponding author.

## Author Contributions

JHL, MD, and OD contributed to the study conception and design. JHL, MD, TR, and OD assisted with data and material collection. JHL and MD performed the data analysis and wrote sections of the manuscript. OD wrote the first draft of the manuscript. All authors contributed to manuscript revision, read, and approved the submitted version.

## Funding

This work was supported by the Finding A Cure for Epilepsy and Seizures (FACES) organization affiliated with NYU Langone Health and the NYU Comprehensive Epilepsy Center. The foundation had no role in study design, data collection and analysis, decision to publish, or preparation of the manuscript.

## Correction note

A correction has been made to this article. Details can be found at: 10.3389/fnut.2025.1635185.

## Conflict of Interest

OD receives grant support from NINDS, NIMH, MURI, CDC, and NSF. He has equity and/or compensation from the following: Tilray, Receptor Life Sciences, Qstate Biosciences, Tevard, Empatica, Engage, Rettco, SilverSpike, and California Cannabis Enterprises (CCE). He has received consulting fees from BridgeBio, Xenon, Zogenix, and Marinus. He holds patents for the use of cannabidiol in treating neurological disorders but these are owned by GW Pharmaceuticals and he has waived any financial stake in these patents. The remaining authors declare that the research was conducted in the absence of any commercial or financial relationships that could be construed as a potential conflict of interest.

## Publisher's Note

All claims expressed in this article are solely those of the authors and do not necessarily represent those of their affiliated organizations, or those of the publisher, the editors and the reviewers. Any product that may be evaluated in this article, or claim that may be made by its manufacturer, is not guaranteed or endorsed by the publisher.
