# Correction: United States dietary trends since 1800: lack of association between saturated fatty acid consumption and non-communicable diseases

**DOI:** 10.3389/fnut.2025.1635185

**Published:** 2025-12-22

**Authors:** Joyce H. Lee, Miranda Duster, Timothy Roberts, Orrin Devinsky

**Affiliations:** 1 Department of Neurology, New York University, Grossman School of Medicine, New York, NY, United States; 2 Medical College of Wisconsin, Milwaukee, WI, United States; 3 New York University, Health Sciences Library, New York, NY, United States

**Keywords:** obesity, diabetes, non-communicable diseases (NCDs), processed foods, nutrients

In the published article, there was an error in the **Introduction**, paragraph 1. The references for citation numbers (1) and (2) were incorrectly referenced as “Tuchman LR. Diabetes, coronary thrombosis and the saccharine disease. Arch Intern Med. (1968) 121:197. doi: 10.1001/archinte.1968.03640020085027 and Ansari I, Grier G, Byers M. Deliberate release: plague - a review. J Biosaf Biosecur. (2020) 2:10–22. doi: 10.1016/j.jobb.2020.02.001.” The corrected references should be written as “Cleave TL, Campbell GD, Painter NS. Diabetes, coronary thrombosis and the saccharine disease. Bristol John Wright (1969) and Bull FC, Al-Ansari SS, Biddle S, Borodulin K, Buman MP, Cardon G, Carty C, Chaput J-P, Chastin S, Chou R, et al. World Health Organization 2020 guidelines on physical activity and sedentary behaviour. Br J Sports Med (2020) 54:1451–1462. doi: 10.1136/bjsports-2020-102955.”

In the published article, there was an error in the **Introduction**, paragraph 2. The citation numbers (2, 3) were incorrectly referenced as “Ansari I, Grier G, Byers M. Deliberate release: plague - a review. J Biosaf Biosecur. (2020) 2:10–22. doi: 10.1016/j.jobb.2020.02.001 and Central Committee for Medical and Community Program of the American Heart Association. Dietary fat and its relation to heart attacks and strokes. J Am Med Assoc. (1961) 175:389–91. doi: 10.1001/jama.1961.63040050001011.” The correct citation number is (3) and the correct reference should have been written as “Keys A. Diet and the epidemiology of coronary heart disease. JAmMed Assoc (1957) 164:1912–1919. doi: 10.1001/jama.1957.62980170024007e.”

In the published article, there was an error in the **Introduction**, paragraph 2. The citation number (4) was incorrectly referenced as “Hirsh J. Revelations from the early annual reports of the Mount Sinai Hospital. J Mt Sinai Hosp N Y. (1953) 20:151–4.”. The correct citation numbers are (4–7) and the correct references should have been written as “Steinberg D. Thematic review series: The Pathogenesis of Atherosclerosis. An interpretive history of the cholesterol controversy: part I1. J Lipid Res (2004) 45:1583–93. doi: 10.1194/jlr.R400003-JLR200; Menotti A, Puddu PE, Kromhout D, Kafatos A, Tolonen H. Coronary heart disease mortality trends during 50 years as explained by risk factor changes: The European cohorts of the Seven Countries Study. Eur J Prev Cardiol (2020) 27:988–98. doi: 10.1177/2047487318821250; Kromhout D, Menotti A, Blackburn H. The Seven Countries Study: A Scientific Adventure in Cardiovascular Disease Epidemiology. Brouwer (1993). Available at: https://books.google.com/books?id=hgWRAAAACAAJ; Keys A. Atherosclerosis: a problem in newer public health. Atherosclerosis (1953) 1:19.”

In the published article, there was an error in the **Introduction**, paragraph 3. The citation number (5) was incorrectly referenced as "Sacks FM, Lichtenstein AH, Wu JHY, Appel LJ, Creager MA, Kris-Etherton PM, et al. Dietary fats and cardiovascular disease: a presidential advisory from the American Heart Association. Circulation. (2017) 136:e1–23. doi: 10.1161/CIR.0000000000000510.” The correct citation number is (8) and the correct reference should have been written as “Association Cc. Dietary Fat and Its Relation to Heart Attacks and Strokes. JAMA (1961) 175:389. doi: 10.1001/jama.1961.63040050001011.”

In the published article, there was an error in the **Introduction**, paragraph 3. The citation numbers (6, 7) were incorrectly referenced as “Hooper L, Martin N, Jimoh OF, Kirk C, Foster E, Abdelhamid AS. Reduction in saturated fat intake for cardiovascular disease. CochraneDatabase Syst Rev. (2020) 2020:11737. doi: 10.1002/14651858.CD011737.pub2; Teng KT, Chang LF, Vethakkan SR, Nesaretnam K, Sanders TAB. Effects of exchanging carbohydrate or monounsaturated fat with saturated fat on inflammatory and thrombogenic responses in subjects with abdominal obesity: a randomized controlled trial. Clin Nutr. (2017) 36:1250–8. doi: 10.1016/j.clnu.2016.08.026.” The correct citation numbers are (9, 10) and the correct references should have been written as “Kannel WB, Gordon T. “The Framingham diet study: Diet and the regulation of serum cholesterol. The Framingham study.,” in An epidemiologic investigation of cardiovascular disease. (Bethesda, MD). Available at: http://www.ncbi.nlm.nih.gov/pubmed/4247338; and Anderson KM, Castelli WP, Levy D. Cholesterol and mortality. 30 years of follow-up from the Framingham study. JAMA (1987) 257:2176–2180. doi: 10.1001/jama.257.16.2176.”

In the published article, there was an error in the **Introduction**, paragraph 3. A reference was missing at the end of this sentence, “In 1977, Senator George McGovern's *Dietary Goals* transformed this hypothesis to national policy and extended SFA's effects to obesity and cancer and recommended reductions for everyone over age 2 years.” This should have been written as “In 1977, Senator George McGovern's Dietary Goals transformed this hypothesis to national policy and extended SFA's effects to obesity and cancer and recommended reductions for everyone over age 2 years (11).” The new reference is “McGovern G. Dietary goals for the United States. Report of the select committee on nutrition and human needs of the United States Senate. (1977).”

In the published article, there was an error in the **Introduction**, paragraph 3. The citation numbers (8–11) were incorrectly referenced as “Dippe SE. Dietary goals for the United States do you agree?. Arizona Med. (1979), 36:587–9; Walker AR, Walker BF. The Surgeon General's Report on Nutrition and Health. Washington, DC: Government Printing Office; Department of Human Services (1989); National Research Council. Diet, Nutrition, and Cancer. Washington, DC: The National Academies Press (1982); American Cancer Society. An american cancer society special report. CA Cancer J Clin. (1984) 34:121–6. doi: 10.3322/canjclin.34.2.121.” The correct citation numbers are (11–14) and the correct references should have been written as “McGovern G. Dietary goals for the United States. Report of the select committee on nutrition and human needs of the United States Senate. (1977); Walker AR, Walker BF. The Surgeon General's Report on Nutrition and Health. ed. D. Services Washington, DC: Government Printing Office (1989). doi: 10.1093/ajcn/50.4.884; National Research Council. Diet, Nutrition, and Cancer. Washington, DC: The National Academies Press (1982). doi: 10.17226/371; American Cancer Society. An American cancer society special report. CA Cancer J Clin. (1984) 34:121–6. doi: 10.3322/canjclin.34.2.121.”

In the published article, there was an error in the **Introduction**, paragraph 3. The citation numbers (12–17) were incorrectly referenced as “Jacobs DR, Blackburn H, Higgins M, Reed D, Iso H, McMillan G, et al. Report of the conference on low blood cholesterol: mortality associations. Circulation. (1992) 86:1046–60. doi: 10.1161/01.CIR.86.3.1046; Williams RR, Sorlie PD, Feinleib M, McNamara PM, Kannel WB, Dawber TR. Cancer incidence by levels of cholesterol. J Am Med Assoc. (1981) 245:247–52. doi: 10.1001/jama.1981.03310280023021; Ravnskov U. The questionable role of saturated and polyunsaturated fatty acids in cardiovascular disease. J Clin Epidemiol. (1998) 51:443–60. doi: 10.1016/S0895-4356(98)00018-3; Binukumar B, Mathew A. Dietary fat and risk of breast cancer. World J Surg Oncol. (2005) 3:22–8. doi: 10.1186/1477-7819-3-45; Zhuang P, Zhang Y, He W, Chen X, Chen J, He L, et al. Dietary fats in relation to total and cause-specific mortality in a prospective cohort of 521 120 individuals with 16 years of followup. Circ Res. (2019) 124:757–68. doi: 10.1161/CIRCRESAHA.118.314038; Ravnskov U. Is saturated fat bad?. In: De Meester F, Zibadi S, Watson RR, editors. Modern Dietary Fat Intakes in Disease Promotion. Nutrition and Health, Part 2. New York, NY: Humana Press (2010). pp. 109–19.” The correct citation numbers are (15–20) and the correct references should have been written as “Jacobs DR, Blackburn H, Higgins M, Reed D, Iso H, McMillan G, et al. Report of the conference on low blood cholesterol: mortality associations. Circulation. (1992) 86:1046–60. doi: 10.1161/01.CIR.86.3.1046; Williams RR, Sorlie PD, Feinleib M, McNamara PM, Kannel WB, Dawber TR. Cancer incidence by levels of cholesterol. J Am Med Assoc. (1981) 245:247–52. doi: 10.1001/jama.1981.03310280023021; Ravnskov U. The questionable role of saturated and polyunsaturated fatty acids in cardiovascular disease. J Clin Epidemiol. (1998) 51:443–60. doi: 10.1016/S0895-4356(98)00018-3; Binukumar B, Mathew A. Dietary fat and risk of breast cancer. World J Surg Oncol. (2005) 3:22–8. doi: 10.1186/1477-7819-3-45; Zhuang P, Zhang Y, He W, Chen X, Chen J, He L, et al. Dietary fats in relation to total and cause-specific mortality in a prospective cohort of 521 120 individuals with 16 years of follow-up. Circ Res. (2019) 124:757–68. doi: 10.1161/CIRCRESAHA.118.314038; Ravnskov U. Is saturated fat bad?. In: De Meester F, Zibadi S, Watson RR, editors. Modern Dietary Fat Intakes in Disease Promotion. Nutrition and Health, Part 2. New York, NY: Humana Press (2010). pp. 109–119.”

In the published article, there was an error in the **Introduction**, paragraph 3. The citation numbers (18, 19) were incorrectly referenced as “Sadeghi A, Shab-Bidar S, Parohan M, Djafarian K. Dietary fat intake and risk of ovarian cancer: a systematic review and dose-response meta-analysis of observational studies. Nutr Cancer. (2019) 71:939–53. doi: 10.1080/01635581.2019.1595049; Ramsden CE, Zamora D, Majchrzak-Hong S, Faurot KR, Broste SK, Frantz RP, et al. Re-evaluation of the traditional diet-heart hypothesis: analysis of Recovered data from Minnesota Coronary Experiment (1968–73). BMJ. (2016) 353:i1246. doi: 10.1136/bmj.i1246.” The correct citation numbers are (21, 22) and the correct references should have been written as “Glade MJ. Food, nutrition, and the prevention of cancer: a global perspective. American Institute for Cancer Research/World Cancer Research Fund, American Institute for Cancer Research, 1997. Nutrition (1999) 15:523–526. doi: 10.1016/s0899-9007(99)00021-0 and Beresford SAA, Johnson KC, Ritenbaugh C, Lasser NL, Snetselaar LG, Black HR, Anderson GL, Assaf AR, Bassford T, Bowen D, et al. Low-fat dietary pattern and risk of colorectal cancer: the Women's Health Initiative Randomized Controlled Dietary Modification Trial. JAMA (2006) 295:643–654. doi: 10.1001/jama.295.6.643.”

In the published article, there was an error in the **Introduction**, paragraph 4. The citation number (20), “Hales CM, Carroll MD, Fryar CD, Ogden CL. Prevalence of obesity and severe obesity among adults: United States, 2017–2018. NCHS Data Brief. (2020) 360:1–8.” was incorrectly numbered. The correct citation number is (23).

In the published article, there was an error in the **Introduction**, paragraph 4. The citation numbers (21, 22) were incorrectly referenced as “McNamara, DJ. The big fat surprise: Why butter, beat and cheese belong in a healthy diet, by nina teicholz. Am. J. Clin. Nutr. (2015) 102:232. doi: 10.3945/ajcn.115.107284; Mann GV, Pearson G, Gordon T, Dawber TR, Lyell L, Shurtleff D. Diet and cardiovascular disease in the Framingham StudyI. Measurement of dietary intake. Am J Clin Nutr. (1962) 11:200–25. doi: 10.1093/ajcn/11.3.200” The correct citation numbers are (9, 24) and the correct references should have been written as “Kannel WB, Gordon T. “The Framingham diet study: Diet and the regulation of serum cholesterol. The Framingham study.,” in An epidemiologic investigation of cardiovascular disease. (Bethesda, MD). Available at: http://www.ncbi.nlm.nih.gov/pubmed/4247338 and Mann GV, Pearson G, Gordon T, Dawber TR, Lyell L, Shurtleff D. Diet and cardiovascular disease in the Framingham StudyI. Measurement of dietary intake. Am J Clin Nutr. (1962) 11:200–25. doi: 10.1093/ajcn/11.3.200.”

In the published article, there was an error in the **Introduction**, paragraph 4. The citation number (23) was incorrectly referenced as “Fredrickson DS, Levy RI, Lees RS. Fat transport in lipoproteins–an integrated approach to mechanisms and disorders. Nutr Rev. (2009) 45:271–3. doi: 10.1111/j.1753-4887.1987.tb02699.x.” The correct citation number is (25) and the correct references should have been written as “Fredrickson DS, Levy RI, Lees RS. Fat transport in lipoproteins-an integrated approach to mechanisms and disorders. Nutr Rev. (2009) 45:271–3. doi: 10.1111/j.1753-4887.1987.tb02699.x.”

In the published article, there was an error in the **Introduction**, paragraph 4. The citation number (24) was incorrectly referenced as “Bhardwaj B, O'Keefe EL, O'Keefe JH. Death by carbs: added sugars and refined carbohydrates cause diabetes and cardiovascular disease in Asian Indians. Mo Med. (2016) 113:395–400.” The correct references (26, 27) are “Sims EA, Bray GA, Danforth E. Experimental obesity in man. VI: The effect of variations in intake of carbohydrate on carbohydrate, lipid, and cortisol metabolism. Horm Metab Res (1974) 6:70–77 and Sims EA, Danforth E, Horton ES. Endocrine and metabolic effects of experimental obesity in man. Recent Prog Horm Res (1973) 29:457–496. doi: 10.1016/b978-0-12-571129-6.50016-6.”

In the published article, there was an error in the **Introduction**, paragraph 5. The citation numbers (8, 25) were incorrectly referenced as “Dippe SE. Dietary goals for the United States do you agree? Arizona Med. (1979), 36: 587–9.554590, and Keys A. Diet and the epidemiology of coronary heart disease. J Am Med Assoc. (1957) 164:1912–9. doi: 10.1001/jama.1957.62980170024007e.” The correct citation numbers are (3, 11) and the correct references should have been written as “Keys A. Diet and the epidemiology of coronary heart disease. J Am Med Assoc. 1957;164(17):1912–9. doi: 10.1001/jama.1957.62980170024007e, and McGovern G. Dietary goals for the United States. Report of the Select Committee on Nutrition and Human Needs of the United States Senate. 1977. Available from: https://ia902204.us.archive.org/32/items/CAT10527234/CAT10527234.pdf”

In the published article, there was an error in the **Introduction**, paragraph 5. The citation numbers (26–28) were incorrectly referenced as “Byers T. Dietary trends in the United States. Relevance to cancer prevention. Cancer. (1993) 72(3 Suppl.):1015–8. doi: 10.1002/1097-0142(19930801)72:3+<1015::AID-CNCR2820721312>3.0.CO;2-Q8334652; Howell S Kones R. “Calories in, calories out” and macronutrient intake: The hope, hype, and science of calories. Am J Physiol Endocrinol Metab. (2017) 313:E608–12. 10.1152/ajpendo.00156.201728765272; and Biener AI Cawley J Meyerhoefer C. The medical care costs of obesity and severe obesity in youth: an instrumental variables approach. Heal Econ. (2020) 29:624–39. 10.1002/hec.400732090412.” The correct citation numbers are (28–30) and the correct references should have been written as “Ramsden CE, Zamora D, Majchrzak-Hong S, Faurot KR, Broste SK, Frantz RP, Davis JM, Ringel A, Suchindran CM, Hibbeln JR. Re-evaluation of the traditional diet-heart hypothesis: analysis of recovered data from Minnesota Coronary Experiment (1968-73). BMJ. 2016 Apr 12;353:i1246. doi: 10.1136/bmj.i1246. Erratum in: BMJ. 2024 Jun 28;385:q1450. doi: 10.1136/bmj.q1450; Schulze MB, Minihane AM, Saleh RNM, Risérus U. Intake and metabolism of omega-3 and omega-6 polyunsaturated fatty acids: nutritional implications for cardiometabolic diseases. Lancet Diabetes Endocrinol. 2020 Nov;8(11):915–30. doi: 10.1016/S2213-8587(20)30148-0; and Howard BV, Van Horn L, Hsia J, Manson JE, Stefanick ML, Wassertheil-Smoller S, et al. Low-fat dietary pattern and risk of cardiovascular disease: the Women's Health Initiative Randomized Controlled Dietary Modification Trial. JAMA. 2006 Feb 8;295(6):655–66. doi: 10.1001/jama.295.6.655.”

In the published article, there was an error in **Materials and Methods**, paragraph 1. The citation numbers (29–34) were incorrectly referenced as “US Department of Agriculture Economic Research Service (ERS). Food Availability (Per Capita) Data System. Available online at: https://www.ers.usda.gov/data-products/food-availability-per-capita-data-system/; US Department of Agriculture. Circular - United States Department of Agriculture. Washington, DC: U.S. Department of Agriculture (1929). Thomas B. Historical Statistics of the United States, 1789–1945: A Supplement to the Statistical Abstract of the United States. Washington, DC (1951). US US Department of Agriculture Bureau Bureau of Agricultural Economics. Consumption of Food in the United States, 1909–52.Washington, DC (1953). p. iv, 249. US US Department of Agriculture Economic Resarch Servic US US Bureau of Agricultural Economics US Agricultural Marketing Service. National Food Situation. Washington, DC: Economic Research Service, U.S. Department of Agriculture (1955). Floud R Fogel RW Harris B Hong SC. The Changing Body: Health, Nutrition, and Human Development in the Western World Since 1700. New York, NY: Cambridge University Press (2011).26413098.” The correct citation numbers are (31–36) and the correct references should have been written as “US Department of Agriculture, Economic Research Service. Food Availability (Per Capita) Data System. Available from: https://www.ers.usda.gov/data-products/food-availability-per-capita-data-system/; US Department of Agriculture. Circular – United States Department of Agriculture. Washington, DC: US Department of Agriculture; 1929. Thomas B. Historical statistics of the United States, 1789–1945: a supplement to the Statistical Abstract of the United States. Washington, DC: US Government Printing Office; 1951. US Department of Agriculture, Bureau of Agricultural Economics. Consumption of food in the United States, 1909–52. Washington, DC: US Department of Agriculture; 1953. p. iv, 249. US Department of Agriculture, Agricultural Marketing Service. The national food situation. NFS-73. Washington, DC: US Department of Agriculture; 1955 Aug 2. Floud R, Fogel RW, Harris B, Hong SC. The changing body: health, nutrition, and human development in the Western world since 1700. New York, NY: Cambridge University Press; 2011.”

In the published article, there was an error in **Materials and Methods**, paragraph 3. The citation numbers (35–43) were incorrectly referenced as “Ross A. Health and diet in 19th-century America: a food historian's point of view. Hist Archaeol. (1993) 27:42–56. 10.1007/BF03374172; Atwater WO Bryant AP. Dietary Studies in New York City in 1896 1897. BiblioBazaar (1902). Available online at: http://hdl.loc.gov/loc.gdc/scd0001.00144244424; Levenstein H. Putting Meat on the American Table: Taste, Technology, Transformation. Baltimore, MD: Johns Hopkins University Press (2006); Kohlmeier AL Cummings RO. The American and his food: a history of food habits in the United States. Mississippi Val Hist Rev. (1941) 28:275. 10.2307/189624812626694; Peters CS. Review: how america eats: A social history of U.S. food and culture. In: WallachJJeditor. Diaìlogo. Vol. 18. Diálogo of the Center for Latino Research at DePaul University (2015). p. 25. Available at: https://via.library.depaul.edu/dialogo/vol18/iss1/25; Carroll A. Three Squares: The Invention of the American Meal. New York, NY: Basic Books (AZ) (2014). Oddy DJ. Review: revolution at the table. In: Levenstein HA editor. The Economic History Review. Vol. 43. Wiley: JSTOR; Economic History Society (1990). pp. 519–20. 10.2307/2596977; Mintz S. Paradox of plenty: A social history of eating in modern america, by Harvey Levenstein. Can. J. Hist. (1993) 28:626–27. 10.3138/cjh.28.3.626.” The correct citation numbers are (37–45) and the correct references should have been written as “Root W, de Rochemont R. Eating in America: a history. New York, NY: William Morrow and Company, Inc.; 1976. Ross A. Health and diet in 19th-century America: a food historian's point of view. Hist Arch. 1993;27:42–56. doi: 10.1007/BF03374172. Atwater WO, Bryant AP. Dietary studies in New York City in 1896–1897. BiblioBazaar; 1902. Available from: https://archive.org/details/dietarystudiesin00atwa; Levenstein H. Putting meat on the American table: taste, technology, transformation. Baltimore, MD: Johns Hopkins University Press; 2006. Cummings RO. The American and his food: a history of food habits in the United States. Revised ed. Chicago: University of Chicago Press; 1940. Available from: https://archive.org/details/americanhisfood0000rich; Wallach JJ. How America eats: a social history of U.S. food and culture. Lanham, MD: Rowman & Littlefield Publishers, Inc.; 2013. Carroll A. Three squares: the invention of the American meal. New York, NY: Basic Books; 2014. Levenstein H. Revolution at the table: the transformation of the American diet. Berkeley: University of California Press; 2003. Levenstein HA. Paradox of plenty: a social history of eating in modern America. New York, NY: Oxford University Press; 1993.”

In the published article, there was an error in **Materials and Methods**, paragraph 5. The citation numbers (29) were incorrectly referenced as “US Department of Agriculture Economic Research Service (ERS). Food Availability (Per Capita) Data System. Available online at: https://www.ers.usda.gov/data-products/food-availability-per-capita-data-system/ “The correct citation number is (31) and the correct reference should have been written as “US Department of Agriculture, Economic Research Service. Food Availability (Per Capita) Data System. Available from: https://www.ers.usda.gov/data-products/food-availability-per-capita-data-system/”

In the published article, there was an error in **Materials and Methods**, paragraph 6. The citation numbers (44, 45) were incorrectly referenced as “Monteiro CA, Cannon G, Moubarac JC, Levy RB, Louzada MLC, Jaime PC. The UN Decade of Nutrition, the NOVA food classification and the trouble with ultra-processing. Public Health Nutr. (2018) 21:5–17. doi: 10.1017/S1368980017000234; Moubarac J-C, Parra DC, Cannon G, Monteiro CA. Food classification systems based on food processing: significance and implications for policies and actions: a systematic literature review and assessment. Curr Obes Rep. (2014) 3:256–72. doi: 10.1007/s13679-014-0092-0.” The correct citation numbers are (46, 47) and the correct references should have been written as “Monteiro CA, Cannon G, Moubarac JC, Levy RB, Louzada MLC, Jaime PC. The UN Decade of Nutrition, the NOVA food classification and the trouble with ultra-processing. Public Health Nutr. 2018 Jan;21(1):5–17. doi: 10.1017/S1368980017000234 and Moubarac JC, Parra DC, Cannon G, Monteiro CA. Food classification systems based on food processing: significance and implications for policies and actions: a systematic literature review and assessment. Curr Obes Rep. 2014 Jun;3(2):256–72. doi: 10.1007/s13679-014-0092-0.”

In the published article, there was an error in **Materials and Methods**, paragraph 8. The citation number (29) was incorrectly referenced as “US Department of Agriculture Economic Research Service (ERS). Food Availability (Per Capita) Data System. Available online at: https://www.ers.usda.gov/data-products/food-availability-per-capita-data-system/”. The correct citation number is (31) and the correct reference should have been written as “US Department of Agriculture, Economic Research Service. Food Availability (Per Capita) Data System. Available from: https://www.ers.usda.gov/data-products/food-availability-per-capita-data-system/”

In the published article, there was an error in **Materials and Methods**, *Data Limitations*, paragraphs 2,3, and 4. The citation number (29) was incorrectly referenced as “US Department of Agriculture Economic Research Service (ERS). Food Availability (Per Capita) Data System. Available online at: https://www.ers.usda.gov/data-products/food-availability-per-capita-data-system/ “The correct citation number is (31) and the correct reference should have been written as “US Department of Agriculture, Economic Research Service. Food Availability (Per Capita) Data System. Available from: https://www.ers.usda.gov/data-products/food-availability-per-capita-data-system/”

In the published article, there was an error in **Materials and Methods**, *Data Limitations*, paragraph 5. The citation number (46) was incorrectly referenced as “The New York Times. Farm Population Lowest Since 1850's. (1988). Available online at: https://www.nytimes.com/1988/07/20/us/farm-population-lowest-since-1850-s.html (accessed July 6, 2021).” The correct citation number is (48) and the correct reference should have been written as “The New York Times. Farm population lowest since 1850's. 1988 Jul 20. Available from: https://www.nytimes.com/1988/07/20/us/farm-population-lowest-since-1850-s.html. Accessed 2021 Jul 6.”

In the published article, there was an error in **Materials and Methods**, *Data Limitations*, paragraph 5. The citation number (47) was incorrectly referenced as “Encyclopaedia Britannica,. Offal. (1998). Available online at: https://www.britannica.com/topic/offal (accessed July 6, 2021).” The correct citation number is (49) and the correct reference should have been written as “Encyclopaedia Britannica. Offal. 1998. Available from: https://www.britannica.com/topic/offal. Accessed 2021 Jul 6.”

In the published article, there was an error in **Materials and Methods**, *Data Limitations*, paragraph 5. The citation number (48) was incorrectly referenced as “Archer E, Thomas DM, McDonald SM, Pavela G, Lavie CJ, Hill JO, et al. The validity of US Nutritional Surveillance: USDA's loss-adjusted food availability data series 1971–2010. Curr Probl Cardiol. (2016) 41:268–92. doi: 10.1016/j.cpcardiol.2016.10.007.” The correct citation number is (50) and the correct reference should have been written as “Archer E, Thomas DM, McDonald SM, Pavela G, Lavie CJ, Hill JO, Blair SN. The validity of US nutritional surveillance: USDA's loss-adjusted food availability data series 1971–2010. Curr Probl Cardiol. 2016 Nov-Dec;41(11, 12):268–92. doi: 10.1016/j.cpcardiol.2016.10.007.”

In the published article, there was an error in **Materials and Methods**, *Data Limitations*, paragraph 5. The citation number (49, 50) were incorrectly referenced as “Putnam JJ Allshouse JE. Food Consumption, Prices, and Expenditures, 1970–97. Washington, DC: U.S. Department of Agriculture, ERS (1999) and Putman J. Major trends in U.S. Food Supply, 1909-99. In: Food Review/ National Food Review. Vol. 23. Washington, DC: United States Department of Agriculture, Economic Research Service (2000). p. 1–8.” The correct citation numbers are (51, 52) and the correct reference should have been written as “Putnam JJ, Allshouse JE. Food consumption, prices, and expenditures, 1970–97. Washington, DC: U.S. Department of Agriculture, Economic Research Service; 1999. Available from: https://www.ers.usda.gov/publications/pub-details?pubid=47115#overview and Putnam J. Major trends in U.S. food supply, 1909–99. Food Rev Natl Food Rev. 2000;23:1–8. Washington, DC: United States Department of Agriculture, Economic Research Service. Available from: https://ageconsearch.umn.edu/record/205508/files/Food%20Review%20Vol.%2023%20Issue%201-2.pdf”

In the published article, there was an error in **Materials and Methods**, *Data Limitations*, paragraph 6. The citation number (29, 48) were incorrectly referenced as “US Department of Agriculture Economic Research Service (ERS). Food Availability (Per Capita) Data System. Available online at: https://www.ers.usda.gov/data-products/food-availability-per-capita-data-system/ and Archer E Thomas DM McDonald SM Pavela G Lavie CJ Hill JO et al. The validity of US Nutritional Surveillance: USDA's loss-adjusted food availability data series 1971-2010. Curr Probl Cardiol. (2016) 41:268–92. 10.1016/j.cpcardiol.2016.10.00727914522.” The correct citation numbers are (31, 50) and the correct reference should have been written as “US Department of Agriculture, Economic Research Service. Food Availability (Per Capita) Data System. Available from: https://www.ers.usda.gov/data-products/food-availability-per-capita-data-system/ and Archer E, Thomas DM, McDonald SM, Pavela G, Lavie CJ, Hill JO, Blair SN. The validity of US nutritional surveillance: USDA's loss-adjusted food availability data series 1971–2010. Curr Probl Cardiol. 2016 Nov-Dec;41(11, 12):268–92. doi: 10.1016/j.cpcardiol.2016.10.007.”

In the published article, there was an error in **Materials and Methods**, *Data Limitations*, paragraph 6. The citation number (51) was incorrectly referenced as “Centers for Disease Control Prevention (CDC) National Center for Health Statistics (NCHS). NHANES - National Health and Nutrition Examination Survey Data. Hyattsville, MD: US Department of Health and Human Services, CDC (2020). Available online at: https://www.cdc.gov/nchs/nhanes/index.htm.” The correct citation number is (53) and the correct reference should have been written as “Centers for Disease Control and Prevention (CDC), National Center for Health Statistics (NCHS). NHANES - National Health and Nutrition Examination Survey Data. Hyattsville, MD: US Department of Health and Human Services, CDC; 2020. Available from: https://www.cdc.gov/nchs/nhanes/index.htm.”

In the published article, there was an error in **Results**, *Added Fats and Oils*, paragraph 5. This sentence previously stated that “One hundred grams of butter contains 848 g of total fat with 45.5 g saturated fatty acids (SFAs), 17 g monounsaturated fatty acids (MUFAs), and 2.5 g polyunsaturated fatty acids (PUFAs), simplified as 848 g/45.5 g/17 g/2.5 g for total fat/SFAs/MUFAs/PUFAs.” This should have been written as “One hundred grams of butter contains 65 g of total fat with 45.5 g saturated fatty acids (SFAs), 17 g monounsaturated fatty acids (MUFAs), and 2.5 g polyunsaturated fatty acids (PUFAs), simplified as 65 g/45.5 g/17 g/2.5 g for total fat/SFAs/MUFAs/PUFAs.”

In the published article, there was an error in **Results**, *Added Fats and Oils*, paragraph 5. The citation number (52) was incorrectly referenced as “US Department of Agriculture Agricultural Research, Service,. FoodData Central. (2019). Available online at: http://fdc.nal.usda.gov/.” The correct citation number is (54) and the correct reference should have been written as “US Department of Agriculture, Agricultural Research Service. FoodData Central. 2019. Available from: http://fdc.nal.usda.gov/.”

In the published article, there was an error in **Results**, *Beverages*, Alcohol, paragraph 1 and 2. The citation number (53) was incorrectly referenced as “Slater ME Alpert SHR. Surveillance Report #117: Apparent Per Capita Alcohol Consumption: National, State, Regional Trends, 1977–2019. (2021). Available online at: https://pubs.niaaa.nih.gov/publications/surveillance117/CONS19.htm (accessed July 21, 2021).” The correct citation number is (55) and the correct reference should have been written as “Slater ME, Alpert SHR. Surveillance report #117: apparent per capita alcohol consumption: national, state, regional trends, 1977–2019. 2021. Available from: https://pubs.niaaa.nih.gov/publications/surveillance117/CONS19.htm. Accessed 2021 Jul 21.”

In the published article, there was an error in **Results**, *Beverages*, Sugar-Sweetened Beverages, paragraph 1. The citation number (54) was incorrectly referenced as “State of Rhode Island Department of Health. Sugar-Sweetened Beverages. Available online at: https://health.ri.gov/healthrisks/sugarsweetenedbeverages/ (accessed July 14, 2021).” The correct citation number is (56) and the correct reference should have been written as “State of Rhode Island Department of Health. Sugar-sweetened beverages. Available from: https://health.ri.gov/healthrisks/sugarsweetenedbeverages/. Accessed 2021 Jul 14.”

In the published article, there was an error in **Results**, *Beverages*, Sugar-Sweetened Beverages, paragraph 1. The citation number (55) was incorrectly referenced as “Bray GA Popkin BM. Dietary sugar and body weight: have we reached a crisis in the epidemic of obesity and diabetes?: health be damned! Pour on the sugar. Diabetes Care. (2014) 37:950–6. 10.2337/dc13-208524652726.” The correct citation number is (57) and the correct reference should have been written as “Bray GA, Popkin BM. Dietary sugar and body weight: have we reached a crisis in the epidemic of obesity and diabetes?: health be damned! Pour on the sugar. Diabetes Care. 2014 Apr;37(4):950–6. doi: 10.2337/dc13-2085.”

In the published article, there was an error in **Results**, *Beverages*, Sugar-Sweetened Beverages, paragraph 2. The citation number (56) was incorrectly referenced as “Duffey KJ Popkin BM. Shifts in patterns and consumption of beverages between 1965 and 2002. Obesity. (2007) 15:2739–47. 10.1038/oby.2007.32618070765.” The correct citation number is (58) and the correct reference should have been written as “Duffey KJ, Popkin BM. Shifts in patterns and consumption of beverages between 1965 and 2002. Obesity (Silver Spring). 2007 Nov;15(11):2739–47. doi: 10.1038/oby.2007.326.”

In the published article, there was an error in **Results**, *Beverages*, Sugar-Sweetened Beverages, paragraph 3. The citation number (57) was incorrectly referenced as “Kit BK Fakhouri THI Park S Nielsen SJ Ogden CL. Trends in sugar-sweetened beverage consumption among youth and adults in the United States: 1999-2010. Am J Clin Nutr. (2013) 98:180–8. 10.3945/ajcn.112.05794323676424.” The correct citation number is (59) and the correct reference should have been written as “Kit BK, Fakhouri TH, Park S, Nielsen SJ, Ogden CL. Trends in sugar-sweetened beverage consumption among youth and adults in the United States: 1999–2010. Am J Clin Nutr. 2013 Jul;98(1):180–8. doi: 10.3945/ajcn.112.057943.”

In the published article, there was an error in **Results**, *Sweetners*, Non-nutritive Sweetners, paragraph 1. The citation number (58) was incorrectly referenced as “Mattes RD Popkin BM. Nonnutritive sweetener consumption in humans: effects on appetite and food intake and their putative mechanisms. Am J Clin Nutr. (2009) 89:1–14. 10.3945/ajcn.2008.2679219056571.” The correct citation number is (60) and the correct reference should have been written as “Mattes RD, Popkin BM. Nonnutritive sweetener consumption in humans: effects on appetite and food intake and their putative mechanisms. Am J Clin Nutr. 2009 Jan;89(1):1–14. doi: 10.3945/ajcn.2008.26792.”

In the published article, there was an error in **Results**, *Sweetners*, Non-nutritive Sweetners, paragraph 2. The citation number (59) was incorrectly referenced as “Dunford EK Miles DR Ng SW Popkin B. Types and amounts of nonnutritive sweeteners purchased by US Households: a comparison of 2002 and 2018 Nielsen homescan purchases. J Acad Nutr Diet. (2020) 120:1662–71.e10. 10.1016/j.jand.2020.04.02232739278.” The correct citation number is (61) and the correct reference should have been written as “Dunford EK, Miles DR, Ng SW, Popkin B. Types and amounts of nonnutritive sweeteners purchased by US households: a comparison of 2002 and 2018 Nielsen Homescan purchases. J Acad Nutr Diet. 2020 Oct;120(10):1662–71.e10. doi: 10.1016/j.jand.2020.04.022.”

In the published article, there was an error in **Results**, *Processed and Ultra Processed Foods*, paragraph 1. The citation number (49) was incorrectly referenced as “Putnam JJ Allshouse JE. Food Consumption, Prices, and Expenditures, 1970-97. Washington, DC: U.S. Department of Agriculture, ERS (1999).” The correct citation number is (51) and the correct reference should have been written as “Putnam JJ, Allshouse JE. Food consumption, prices, and expenditures, 1970–97. Washington, DC: U.S. Department of Agriculture, Economic Research Service; 1999. Available from: https://www.ers.usda.gov/publications/pub-details?pubid=47115#overview”

In the published article, there was an error in **Results**, *Processed and Ultra Processed Foods*, paragraph 1. The citation number (35) were incorrectly referenced as “Root W de Rochemont R. Eating in America, A History. New York, NY: William Morrow and Company, Inc. (1976).” The correct citation number is (37) and the correct reference should have been written as “Root W, de Rochemont R. Eating in America: a history. New York, NY: William Morrow and Company, Inc.; 1976.”

In the published article, there was an error in **Results**, *Processed and Ultra Processed Foods*, Processed Meat paragraph 1. The citation number (60) was incorrectly referenced as “Zeng L Ruan M Liu J Wilde P Naumova EN Mozaffarian D et al. Trends in processed meat, unprocessed red meat, poultry, and fish consumption in the United States, 1999-2016. J Acad Nutr Diet. (2019) 119:1085–98.e12. 10.1016/j.jand.2019.04.00431234969.” The correct citation number is (62) and the correct reference should have been written as “Zeng L, Ruan M, Liu J, Wilde P, Naumova EN, Mozaffarian D, Zhang FF. Trends in processed meat, unprocessed red meat, poultry, and fish consumption in the United States, 1999–2016. J Acad Nutr Diet. 2019 Jul;119(7):1085–98.e12. doi: 10.1016/j.jand.2019.04.004.”

In the published article, there was an error in **Results**, *Processed and Ultra Processed Foods*, Processed Cereal paragraph 1. The citation numbers (61–65) were incorrectly referenced as “Stiebeling HK Monroe D Coons CM Phipard EF Clark F. Family Economics Division Bureau of Home Economics. CONSUMER PURCHASES STUDY Urban Village Series Family Food Consumption Dietary Levels Five Regions. (1941). Available online at: https://www.ars.usda.gov/ARSUserFiles/80400530/pdf/hist/bhe_1941_misc_pub_452.pdf (accessed July 21, 2021). Stiebeling HK Monroe D Coons CM Phipard EF Clark F. Family Economics Division Bureau of Home Economics. CONSUMER PURCHASES STUDY Farm Series Family Food Consumption Dietary Levels Five Regions. (1941). Available online at: https://www.ars.usda.gov/ARSUserFiles/80400530/pdf/hist/bhe_1941_misc_pub_405.pdf (accessed July 21, 2021). US Department of Agriculture. Family Food Consumption in the United States (Miscellaneous Publication No. 550). (1944). Available online at: https://www.ars.usda.gov/ARSUserFiles/80400530/pdf/hist/bhnhe_1944_misc_pub_550.pdf (accessed July 21, 2021). US Department of Agriculture. Food Consumption of Households in the United States (Household Food Consumption Survey 1955 Report No. 1). Washington, D.C. Available online at: https://www.ars.usda.gov/ARSUserFiles/80400530/pdf/5556/hfcs5556_rep_1.pdf (accessed July 21, 2021). US Department of Agriculture. Household Food Consumption Survey 1965-66. Available online at: https://www.ars.usda.gov/ARSUserFiles/80400530/pdf/6566/hfcs6566_rep_12.pdf (accessed July 21, 2021).” The correct citation numbers are (63–67) and the correct references should have been written as “Stiebeling HK, Monroe D, Coons CM, Phipard EF, Clark F. Consumer purchases study: urban village series family food consumption dietary levels five regions. Washington, DC: Family Economics Division, Bureau of Home Economics; 1941. Available from: https://www.ars.usda.gov/ARSUserFiles/80400530/pdf/hist/bhe_1941_misc_pub_452.pdf. Accessed 2021 Jul 21. Stiebeling HK, Monroe D, Coons CM, Phipard EF, Clark F. Consumer purchases study: farm series family food consumption dietary levels five regions. Washington, DC: Family Economics Division, Bureau of Home Economics; 1941. Available from: https://www.ars.usda.gov/ARSUserFiles/80400530/pdf/hist/bhe_1941_misc_pub_405.pdf. Accessed 2021 Jul 21. US Department of Agriculture. Family food consumption in the United States (Miscellaneous Publication No. 550). Washington, DC: US Department of Agriculture; 1944. Available from: https://www.ars.usda.gov/ARSUserFiles/80400530/pdf/hist/bhnhe_1944_misc_pub_550.pdf. Accessed 2021 Jul 21. US Department of Agriculture. Food consumption of households in the United States (Household Food Consumption Survey 1955 Report No. 1). Washington, DC: US Department of Agriculture; 1956. Available from: https://www.ars.usda.gov/ARSUserFiles/80400530/pdf/5556/hfcs5556_rep_1.pdf. Accessed 2021 Jul 21. US Department of Agriculture. Household Food Consumption Survey 1965-66. Washington, DC: US Department of Agriculture; 1972. Available from: https://www.ars.usda.gov/ARSUserFiles/80400530/pdf/6566/hfcs6566_rep_12.pdf. Accessed 2021 Jul 21.”

In the published article, there was an error in **Results**, *Processed and Ultra Processed Foods*, Processed Cereal paragraph 1. The citation number (66) was incorrectly referenced as “Smith BJ Yonkers RD. The Importance of Cereal to Fluid Milk Consumption. AE & RS Research Reports 257707. Pennsylvania State University, Department of Agricultural Economics and Rural Sociology (1990). 10.22004/ag.econ.257707a” The correct citation number is (68) and the correct reference should have been written as “Smith BJ, Yonkers RD. The importance of cereal to fluid milk consumption. AE & RS Research Reports 257707. Pennsylvania State University, Department of Agricultural Economics and Rural Sociology; 1990. Available from: https://ageconsearch.umn.edu/record/257707/files/magr-pennstate-035.pdf.”

In the published article, there was an error in **Results**, *Processed and Ultra Processed Foods*, Processed Cereal paragraph 1. The citation number (50) was incorrectly referenced as “Putman J. Major trends in U.S. Food Supply, 1909-99. In: Food Review/ National Food Review. Vol. 23. Washington, DC: United States Department of Agriculture, Economic Research Service (2000). p. 1–8.” The correct citation number is (52) and the correct reference should have been written as “Putnam J. Major trends in U.S. food supply, 1909–99. Food Rev Natl Food Rev. 2000;23:1–8. Washington, DC: United States Department of Agriculture, Economic Research Service. Available from: https://ageconsearch.umn.edu/record/205508/files/Food%20Review%20Vol.%2023%20Issue%201-2.pdf.”

In the published article, there was an error in **Results**, *Processed and Ultra Processed Foods*, Processed Cereal paragraph 2. The citation number (67) was incorrectly referenced as “Zhu Y Jain N Vanage V Holschuh N Agler AH Smith JD. Association between ready-to-eat cereal consumption and nutrient intake, nutritional adequacy, and diet quality in adults in the National Health and Nutrition Examination Survey 2015-2016. Nutrients. (2019) 11:2952. 10.3390/nu1112295231817088.” The correct citation number is (69) and the correct reference should have been written as “Zhu Y, Jain N, Vanage V, Holschuh N, Agler AH, Smith JD. Association between ready-to-eat cereal consumption and nutrient intake, nutritional adequacy, and diet quality in adults in the National Health and Nutrition Examination Survey 2015–2016. Nutrients. 2019 Dec 4;11(12):2952. doi: 10.3390/nu11122952.”

In the published article, there was an error in **Results**, *Processed and Ultra Processed Foods*, Processed Cereal paragraph 3. The citation number (68) was incorrectly referenced as “Smith JD Zhu Y Vanage V Jain N Holschuh N Hermetet Agler A. Association between ready-to-eat cereal consumption and nutrient intake, nutritional adequacy, and diet quality among infants, toddlers, and children in the national health and nutrition examination survey 2015-2016. Nutrients. (2019) 11:1989. 10.3390/nu1109198931443588.” The correct citation number is (70) and the correct reference should have been written as “Smith JD, Zhu Y, Vanage V, Jain N, Holschuh N, Hermetet Agler A. Association between ready-to-eat cereal consumption and nutrient intake, nutritional adequacy, and diet quality among infants, toddlers, and children in the National Health and Nutrition Examination Survey 2015–2016. Nutrients. 2019 Aug 23;11(9):1989. doi: 10.3390/nu11091989.”

In the published article, there was an error in **Discussion**, paragraph 1. The citation number (44) was incorrectly referenced as “Monteiro CA Cannon G Moubarac JC Levy RB Louzada MLC Jaime PC. The UN Decade of Nutrition, the NOVA food classification and the trouble with ultra-processing. Public Health Nutr. (2018) 21:5–17. 10.1017/S136898001700023428322183.” The correct citation number is (46) and the correct reference should have been written as “Monteiro CA, Cannon G, Moubarac JC, Levy RB, Louzada MLC, Jaime PC. The UN Decade of Nutrition, the NOVA food classification and the trouble with ultra-processing. Public Health Nutr. 2018 Jan;21(1):5–17. doi: 10.1017/S1368980017000234.”

In the published article, there was an error in **Discussion**, paragraph 2. The citation numbers (8, 25, 69, 70) were incorrectly referenced as “Dippe SE. Dietary goals for the United States do you agree?. Arizona Med. (1979), 36: 587–9.554590; Keys A. Diet and the epidemiology of coronary heart disease. J Am Med Assoc. (1957) 164:1912–9. 10.1001/jama.1957.62980170024007e13448947; Koop CE. The Surgeon General's Report on Nutrition Health. US Department Health Human Services. DHSS Pub # 88-50210 (1998). Available online at: https://profiles.nlm.nih.gov/101584932X695; Brewster L Jacobson MF. The Changing American Diet. Washington, DC: Center for Science in the Public Interest (1978). p. 80.” The correct citation numbers are (3, 11, 22, 71) and the correct references should have been written as “Keys A. Diet and the epidemiology of coronary heart disease. J Am Med Assoc. 1957;164(17):1912–9. doi: 10.1001/jama.1957.62980170024007e; McGovern G. Dietary goals for the United States. Report of the Select Committee on Nutrition and Human Needs of the United States Senate. 1977. Available from: https://ia902204.us.archive.org/32/items/CAT10527234/CAT10527234.pdf; Koop CE, Nestle M. The Surgeon General's report on nutrition and health. Washington, DC: U.S. Department of Health and Human Services, Public Health Service, Office of the Surgeon General; 1988. Available from: https://profiles.nlm.nih.gov/101584932X695; Brewster L, Jacobson MF. The changing American diet. Washington, DC: Center for Science in the Public Interest; 1978.”

In the published article, there was an error in **Discussion**, paragraph 3. The citation numbers (48, 71–75) were incorrectly referenced as “Archer E Thomas DM McDonald SM Pavela G Lavie CJ Hill JO et al. The validity of US Nutritional Surveillance: USDA's loss-adjusted food availability data series 1971-2010. Curr Probl Cardiol. (2016) 41:268–92. doi: 10.1016/j.cpcardiol.2016.10.00727914522; Archer E Hand GA Blair SN. Validity of U.S. nutritional surveillance:National Health and Nutrition Examination Survey caloric energy intake data, 1971-201. PLoS ONE. (2013) 8:e76632. doi: 10.1371/journal.pone.007663224130784; Schoeller DA. Limitations in the assessment of dietary energy intake by self-report. Metabolism. (1995) 44:18–22. doi: 10.1016/0026-0495(95)90204-X7869932; Subar AF Kipnis V Troiano RP Midthune D Schoeller DA Bingham S et al. Using intake biomarkers to evaluate the extent of dietary misreporting in a large sample of adults: The OPEN study. Am J Epidemiol. (2003) 158:1–13. doi: 10.1093/aje/kwg09212835280; Ioannidis JPA. Implausible results in human nutrition research. BMJ. (2013) 347:f6698. doi: 10.1136/bmj.f669824231028; Brody JE. Good Food Book: Living the High-Carbohydrate Way. New York, NY: W. W. Norton & Company (1985). p. 700.” The correct citation numbers are (50, 73–77) and the correct references should have been written as “Archer E, Thomas DM, McDonald SM, Pavela G, Lavie CJ, Hill JO, Blair SN. The validity of US nutritional surveillance: USDA's loss-adjusted food availability data series 1971–2010. Curr Probl Cardiol. 2016 Nov-Dec;41(11, 12):268–92. doi: 10.1016/j.cpcardiol.2016.10.007; Archer E, Hand GA, Blair SN. Validity of U.S. nutritional surveillance: National Health and Nutrition Examination Survey caloric energy intake data, 1971–2010. PLoS One. 2013 Oct 9;8(10):e76632. doi: 10.1371/journal.pone.0076632. Erratum in: PLoS One. 2013 Oct 11;8(10). doi: 10.1371/annotation/c313df3a-52bd-4cbe-af14-6676480d1a43; Schoeller DA. Limitations in the assessment of dietary energy intake by self-report. Metabolism. 1995 Feb;44(2 Suppl 2):18–22. doi: 10.1016/0026-0495(95)190204-x; Subar AF, Kipnis V, Troiano RP, Midthune D, Schoeller DA, Bingham S, et al. Using intake biomarkers to evaluate the extent of dietary misreporting in a large sample of adults: the OPEN study. Am J Epidemiol. 2003 Jul 1;158(1):1–13. doi: 10.1093/aje/kwg092; Ioannidis JP. Implausible results in human nutrition research. BMJ. 2013 Nov 14;347:f6698. doi: 10.1136/bmj.f6698; Brody JE. Jane Brody's good food book: living the high-carbohydrate way. Illustrated ed. New York, NY: W. W. Norton & Company; 1985.”

In the published article, there was an error in **Discussion**, *The Changing American Diet: History and Influence*, paragraph 1. The citation numbers (8, 69, 70, 75, 76) were incorrectly referenced as “Dippe SE. Dietary goals for the United States do you agree?. Arizona Med. (1979), 36: 587–9.554590; Koop CE. The Surgeon General's Report on Nutrition Health. US Department Health Human Services. DHSS Pub # 88-50210 (1998). Available online at: https://profiles.nlm.nih.gov/101584932X695; Brewster L Jacobson MF. The Changing American Diet. Washington, DC: Center for Science in the Public Interest (1978). p. 80; Brody JE. Good Food Book: Living the High-Carbohydrate Way. New York, NY: W.W. Norton & Company (1985). p. 700; World Health Organization. Diet, Nutrition, and the Prevention of Chronic Diseases: Report of a Joint WHO/FAO Expert Consultation. Geneva: World Health Organization (2003). p. 149.” The correct citation numbers are (11, 71, 72, 78) and the correct references should have been written as “McGovern G. Dietary goals for the United States. Report of the Select Committee on Nutrition and Human Needs of the United States Senate. 1977. Available from: https://ia902204.us.archive.org/32/items/CAT10527234/CAT10527234.pdf; Koop CE, Nestle M. The Surgeon General's report on nutrition and health. Washington, DC: U.S. Department of Health and Human Services, Public Health Service, Office of the Surgeon General; 1988. Available from: https://profiles.nlm.nih.gov/101584932X695; Brewster L, Jacobson MF. The changing American diet. Washington, DC: Center for Science in the Public Interest; 1978; World Health Organization. Diet, nutrition, and the prevention of chronic diseases: report of a joint WHO/FAO expert consultation. Geneva: World Health Organization; 2003. Available from: https://iris.who.int/bitstream/handle/10665/42665/WHO_TRS_916.pdf?sequence=1.”

In the published article, there was an error in **Discussion**, *The Changing American Diet: History and Influence*, paragraph 1. The citation number (77) was incorrectly referenced as “Gebhardt SE Lemar LE Pehrsson PR Exler J Haytowitz DB Patterson KK. USDA National Nutrient Database for Standard Reference, Release 22. USDA National Nutrient Database for Standard Reference.ARS, USDA: Nutrient Data Laboratory, Beltsville Human Nutrition Research Center (2007).22439632.” The correct citation number is (79) and the correct reference should have been written as “U.S. Department of Agriculture, Agricultural Research Service, USDA Nutrient Data Laboratory. USDA National Nutrient Database for Standard Reference, Release 22. 2009. Available from: https://www.ars.usda.gov/ARSUserFiles/80400535/DATA/sr22/sr22_doc.pdf.”

In the published article, there was an error in **Discussion**, *The Changing American Diet: History and Influence*, paragraph 1. The citation number (78) was incorrectly referenced as “Heini AF, Weinsier RL. Divergent trends in obesity and fat intake patterns: the American paradox. Am J Med. (1997) 102:259–64. doi: 10.1016/S0002-9343(96)00456-19217594.” The correct citation number is (80) and the correct reference should have been written as “Heini AF, Weinsier RL. Divergent trends in obesity and fat intake patterns: the American paradox. Am J Med. 1997 Mar;102(3):259-64. doi: 10.1016/S0002-9343(96)00456-1.”

In the published article, there was an error in **Discussion**, *The Changing American Diet: History and Influence*, paragraph 2. The citation number (8) was incorrectly referenced as “Dippe SE. Dietary goals for the United States do you agree?. Arizona Med. (1979), 36: 587–9.554590.” The correct citation number is (11) and the correct reference should have been written as “McGovern G. Dietary goals for the United States. Report of the Select Committee on Nutrition and Human Needs of the United States Senate. 1977. Available from: https://ia902204.us.archive.org/32/items/CAT10527234/CAT10527234.pdf”

In the published article, there was an error in **Discussion**, *The Changing American Diet: History and Influence*, paragraph 2. The citation number (79) was incorrectly referenced as “Schneeman BO. The dietary guidelines for Americans. J Am Diet Assoc. (2001) 101:742–3. 10.1016/S0002-8223(01)00183-311478467.” The correct citation number is (81) and the correct reference should have been written as “Schneeman BO. The Dietary Guidelines for Americans: a basis for US nutrition policy. J Am Diet Assoc. 2001 Jul;101(7):742–3. doi: 10.1016/S0002-8223(01)00183-3.”

In the published article, there was an error in **Discussion**, *The Changing American Diet: History and Influence*, paragraph 2. The citation number (76) was incorrectly referenced as “World Health Organization. Diet, Nutrition, and the Prevention of Chronic Diseases: Report of a Joint WHO/FAO Expert Consultation. Geneva: World Health Organization (2003). p. 149.” The correct citation number is (80) and the correct reference should have been written as “Heini AF, Weinsier RL. Divergent trends in obesity and fat intake patterns: the American paradox. Am J Med. 1997 Mar;102(3):259-64. doi: 10.1016/S0002-9343(96)00456-1.”

In the published article, there was an error in **Discussion**, *The Changing American Diet: History and Influence*, paragraph 3. The citation number (80) was incorrectly referenced as “Kannel WB Gordon T. The Framingham diet study: diet and the regulation of serum cholesterol. In: The Framingham study: An Epidemiologic Investigation of Cardiovascular Disease. Section 24. Washington, DC: U.S. Department of Health, Education, and Welfare, National Institutes of Health (1971) 11976156.” The correct citation number is (9) and the correct reference should have been written as “Gordon T, Kannel WB. Framingham Study. Framingham, MA: Framingham Heart Study; 1968 Jun. Available from: https://archive.org/details/framinghamstudye00kann_22/page/n3/mode/2up”

In the published article, there was an error in **Discussion**, *The Changing American Diet: History and Influence*, paragraph 3. The citation number (81) was incorrectly referenced as “Gordon T Castelli WP Hjortland MC Kannel WB Dawber TR. High density lipoprotein as a protective factor against coronary heart disease. The Framingham study. Am J Med. (1977) 62:707–14. doi: 10.1016/0002-9343(77)90874-9193398.” The correct citation number is (82) and the correct reference should have been written as “Gordon T, Castelli WP, Hjortland MC, Kannel WB, Dawber TR. High density lipoprotein as a protective factor against coronary heart disease. The Framingham Study. Am J Med. 1977 May;62(5):707-14. doi: 10.1016/0002-9343(77)90874-9.

In the published article, there was an error in **Discussion**, *The Changing American Diet: History and Influence*, paragraph 3. The citation numbers (82, 83) were incorrectly referenced as “Fredrickson DS Lees RS. A system for phenotyping hyperlipoproteinemia. Circulation. (1965) 31:321–7. doi: 10.1161/01.CIR.31.3.32114262568 and Volek JS Fernandez ML Feinman RD Phinney SD. Dietary carbohydrate restriction induces a unique metabolic state positively affecting atherogenic dyslipidemia, fatty acid partitioning, and metabolic syndrome. Prog Lipid Res. (2008) 47:307–18. 10.1016/j.plipres.2008.02.00318396172.” The correct citation number were (83, 84) and the correct references should have been written as “Fredrickson DS, Lees RS. A system for phenotyping hyperlipoproteinemia. Circulation. 1965 Mar;31:321-7. doi: 10.1161/01.cir.31.3.321 and Volek JS, Fernandez ML, Feinman RD, Phinney SD. Dietary carbohydrate restriction induces a unique metabolic state positively affecting atherogenic dyslipidemia, fatty acid partitioning, and metabolic syndrome. Prog Lipid Res. 2008 Sep;47(5):307–18. doi: 10.1016/j.plipres.2008.02.003.”

In the published article, there was an error in **Discussion**, *The Changing American Diet: History and Influence*, paragraph 3. The citation number (84, 85) were incorrectly referenced as “Franco OH Peeters A Bonneux L De Laet C. Blood pressure in adulthood and life expectancy with cardiovascular disease in men and women: Life course analysis. Hypertension. (2005) 46:280–6. 10.1161/01.HYP.0000173433.67426.9b15983235 and Gordon T Dawber TR Hjortland MC Kannel WB. Predicting coronary heart disease in middle-aged and older persons: the Framington study. J Am Med Assoc. (1977) 238:497–9. 10.1001/jama.238.6.497577575.” The correct citation number were (85, 86) and the correct references should have been written as “Franco OH, Peeters A, Bonneux L, de Laet C. Blood pressure in adulthood and life expectancy with cardiovascular disease in men and women: life course analysis. Hypertension. (2005) 46:280–6. doi: 10.1161/01.HYP.0000173433.67426.9b and Gordon T, Castelli WP, Hjortland MC, Kannel WB, Dawber TR. Predicting coronary heart disease in middle-aged and older persons: The Framingham study. JAMA. (1977) 238:497–9. PMID: 577575.”

In the published article, there was an error in **Discussion**, *The Changing American Diet: History and Influence*, paragraph 3. The citation number (80) was incorrectly referenced as “Kannel WB Gordon T. The Framingham diet study: diet and the regulation of serum cholesterol. In: The Framingham study: An Epidemiologic Investigation of Cardiovascular Disease. Section 24. Washington, DC: U.S. Department of Health, Education, and Welfare, National Institutes of Health (1971).11976156.” The correct citation number is (9) and the correct reference should have been written as “Gordon T, Kannel WB. Framingham Study. Framingham, MA: Framingham Heart Study; 1968 Jun. Available from: https://archive.org/details/framinghamstudye00kann_22/page/n3/mode/2up”

In the published article, there was an error in **Discussion**, *The Changing American Diet: History and Influence*, paragraph 3. The citation numbers (19, 86–89) were incorrectly referenced as “Ramsden CE Zamora D Majchrzak-Hong S Faurot KR Broste SK Frantz RP et al. Re-evaluation of the traditional diet-heart hypothesis: analysis of Recovered data from Minnesota Coronary Experiment (1968-73). BMJ. (2016) 353:i1246. doi: 10.1136/bmj.i124627071971; Kuulasmaa K Tunstall-Pedoe H Dobson A Fortmann S Sans S Tolonen H et al. Estimation of contribution of changes in classic risk factors to trends in coronary-event rates across the WHO MONICA Project populations. Lancet. (2000) 355:675–87. doi: 10.1016/S0140-6736(99)11180-210703799; Kjelsberg MO. Multiple risk factor intervention trial: risk factor changes and mortality results. J Am Med Assoc. (1982) 248:1465–77. doi: 10.1001/jama.248.12.14659032168; Howard B V Van Horn L Hsia J Manson JAE Stefanick ML Wassertheil-Smoller S et al. Low-fat dietary pattern and risk of cardiovascular disease: the Women's Health Initiative randomized controlled dietary modification trial. J Am Med Assoc. (2006) 295:655–66. doi: 10.1001/jama.295.6.65518377781; Christakis G Rinzler SH Archer M Winslow G Jampel S Stephenson J et al. The anti-coronary club. A dietary approach to the prevention of coronary heart disease–a seven-year report. Am J Public Health Nations Health. (1966) 56:299–314. doi: 10.2105/AJPH.56.2.2995948223.” The correct citation numbers are (28, 30, 87–89) and the correct references should have been written as “Ramsden CE, Zamora D, Majchrzak-Hong S, Faurot KR, Broste SK, Frantz RP, Davis JM, Ringel A, Suchindran CM, Hibbeln JR. Re-evaluation of the traditional diet-heart hypothesis: analysis of recovered data from Minnesota Coronary Experiment (1968-73). BMJ. 2016 Apr 12;353:i1246. doi: 10.1136/bmj.i1246. Erratum in: BMJ. 2024 Jun 28;385:q1450. doi: 10.1136/bmj.q1450; Howard BV, Van Horn L, Hsia J, Manson JE, Stefanick ML, Wassertheil-Smoller S, et al. Low-fat dietary pattern and risk of cardiovascular disease: the Women's Health Initiative Randomized Controlled Dietary Modification Trial. JAMA. 2006 Feb 8;295(6):655–66. doi: 10.1001/jama.295.6.655; Kuulasmaa K, Tunstall-Pedoe H, Dobson A, Fortmann S, Sans S, Tolonen H, et al. Estimation of contribution of changes in classic risk factors to trends in coronary-event rates across the WHO MONICA Project populations. Lancet. 2000 Feb 26;355(9205):675-87. doi: 10.1016/s0140-6736(99)11180-2; The Multiple Risk Factor Intervention Trial Research Group. Multiple Risk Factor Intervention Trial: Risk Factor Changes and Mortality Results. JAMA. 1982 Sep 24;248(12):1465-77. doi: 10.1001/jama.1982.03330120023025; Christakis G, Rinzler SH, Archer M, Winslow G, Jampel S, Stephenson J, et al. The anti-coronary club. A dietary approach to the prevention of coronary heart disease—a seven-year report. Am J Public Health Nations Health. 1966 Feb;56(2):299-314. doi: 10.2105/ajph.56.2.299.”

In the published article, there was an error in **Discussion**, *The Changing American Diet: History and Influence*, paragraph 4. The citation number (9) was incorrectly cited at the end of this sentence “McGovern's Senate Select Committee's Dietary Goals for America (1977) was pivotal in definitively linking dietary SFAs as a major cause of heart disease, obesity, and cancer.” There should not be any citations there.

In the published article, there was an error in **Discussion**, *The Changing American Diet: History and Influence*, paragraph 4. The citation number (90) was incorrectly referenced as “Dietary goals for the United States: statement of The American Medical Association to the Select Committee on Nutrition and Human Needs United States Senate. R I Med J. (1977) 60:576–81.” The correct citation number is (11) and the correct references should have been written as “McGovern G. Dietary goals for the United States. Report of the Select Committee on Nutrition and Human Needs of the United States Senate. 1977. Available from: https://ia902204.us.archive.org/32/items/CAT10527234/CAT10527234.pdf.”

In the published article, there was an error in **Discussion**, *The Changing American Diet: History and Influence*, paragraph 4. The citation number (23) was incorrectly referenced as “Fredrickson DS Levy RI Lees RS. Fat transport in lipoproteins-an integrated approach to mechanisms and disorders. Nutr Rev. (2009) 45:271–3. 10.1111/j.1753-4887.1987.tb02699.x3331729.” The correct citation number is (25) and the correct reference should have been written as “Fredrickson DS, Levy RI, Lees RS. Fat transport in lipoproteins—an integrated approach to mechanisms and disorders. Nutr Rev. 1987 Nov;45(11):271–3. doi: 10.1111/j.1753-4887.1987.tb02699.x.”

In the published article, there was an error in **Discussion**, *The Changing American Diet: History and Influence*, paragraph 5. The citation number (91) was incorrectly referenced as “Bantle JP. Nutrition recommendations and interventions for diabetes: A position statement of the American Diabetes Association (Diabetes Care (2008) 31, Suppl. 1, (S61-S78)). Diabetes Care. (2010) 33:1911. 10.2337/dc08-S06118165339.” The correct citation number is (90) and the correct reference should have been written as “American Diabetes Association; Bantle JP, Wylie-Rosett J, Albright AL, Apovian CM, Clark NG, Franz MJ, et al. Nutrition recommendations and interventions for diabetes: a position statement of the American Diabetes Association. Diabetes Care. 2008 Jan;31 Suppl 1:S61-78. doi: 10.2337/dc08-S061. Erratum in: Diabetes Care. 2010 Aug;33(8):1911.”

In the published article, there was an error in **Discussion**, *The Changing American Diet: History and Influence*, paragraph 5. The citation numbers (19, 88, 92, 93) were incorrectly referenced as “Ramsden CE Zamora D Majchrzak-Hong S Faurot KR Broste SK Frantz RP et al. Re-evaluation of the traditional diet-heart hypothesis: analysis of Recovered data from Minnesota Coronary Experiment (1968-73). BMJ. (2016) 353:i1246. doi: 10.1136/bmj.i124627071971; Howard B V Van Horn L Hsia J Manson JAE Stefanick ML Wassertheil-Smoller S et al. Low-fat dietary pattern and risk of cardiovascular disease: the Women's Health Initiative randomized controlled dietary modification trial. J Am Med Assoc. (2006) 295:655–66. doi: 10.1001/jama.295.6.65518377781; Fung TT Rexrode KM Mantzoros CS Manson JE Willett WC Hu FB. Mediterranean diet and incidence of and mortality from coronary heart disease and stroke in women. Circulation. (2009) 119:1093–100. doi: 10.1161/CIRCULATIONAHA.108.81673619221219 and Schulze MB Minihane AM Noureldin R Saleh M Risérus U Schulze M. Review Intake and metabolism of n-3 and n-6 PUFA: Nutritional implications for cardiometabolic diseases. Lancet Diabetes Endocrinol. (2020) 11:915–30. doi: 10.1016/S2213-8587(20)30148-032949497.” The correct citation numbers are (28–30, 91) and the correct reference should have been written as “Ramsden CE, Zamora D, Majchrzak-Hong S, Faurot KR, Broste SK, Frantz RP, Davis JM, Ringel A, Suchindran CM, Hibbeln JR. Re-evaluation of the traditional diet-heart hypothesis: analysis of recovered data from Minnesota Coronary Experiment (1968-73). BMJ. 2016 Apr 12;353:i1246. doi: 10.1136/bmj.i1246. Erratum in: BMJ. 2024 Jun 28;385:q1450. doi: 10.1136/bmj.q1450; Schulze MB, Minihane AM, Saleh RNM, Risérus U. Intake and metabolism of omega-3 and omega-6 polyunsaturated fatty acids: nutritional implications for cardiometabolic diseases. Lancet Diabetes Endocrinol. 2020 Nov;8(11):915–30. doi: 10.1016/S2213-8587(20)30148-0; Howard BV, Van Horn L, Hsia J, Manson JE, Stefanick ML, Wassertheil-Smoller S, et al. Low-fat dietary pattern and risk of cardiovascular disease: the Women's Health Initiative Randomized Controlled Dietary Modification Trial. JAMA. 2006 Feb 8;295(6):655–66. doi: 10.1001/jama.295.6.655 and Fung TT, Rexrode KM, Mantzoros CS, Manson JE, Willett WC, Hu FB. Mediterranean diet and incidence of and mortality from coronary heart disease and stroke in women. Circulation. 2009;119(8):1093–100. doi: 10.1161/CIRCULATIONAHA.108.816736.”

In the published article, there was an error in **Discussion**, *The Changing American Diet: History and Influence*, paragraph 5. The citation number (94–96) were incorrectly referenced as “World Health Organization. The world health report 2001-mental health: new understanding, new hope. Bull World Health Organ. (2001) 79:1085; Siri-Tarino PW Chiu S Bergeron N Krauss RM. Saturated fats versus polyunsaturated fats versus carbohydrates for cardiovascular disease prevention and treatment. Annu Rev Nutr. (2015) 35:517–43. doi: 10.1146/annurev-nutr-071714-03444926185980 and Kris-Etherton PM Pearson TA Wan Y Hargrove RL Moriarty K Fishell V et al. High-monounsaturated fatty acid diets lower both plasma cholesterol and triacylglycerol concentrations. Am J Clin Nutr. (1999) 70:1009–15. doi: 10.1093/ajcn/70.6.100910584045.” The correct citation numbers are (92, 93) and the correct reference should have been written as “Siri-Tarino PW, Chiu S, Bergeron N, Krauss RM. Saturated fats versus polyunsaturated fats versus carbohydrates for cardiovascular disease prevention and treatment. Annu Rev Nutr. 2015;35:517-43. doi: 10.1146/annurev-nutr-071714-034449 and World Health Organization. The World health report: 2001: Mental health: new understanding, new hope. Geneva: World Health Organization; 2001. Available from: https://iris.who.int/handle/10665/42390.”

In the published article, there was an error in **Discussion**, *The Changing American Diet: History and Influence*, paragraph 6. The citation number (94) was incorrectly cited at the end of this sentence, “The profound dietary changes were accompanied by other lifestyle and demographic changes, including (1) increased urbanization and population density, (2) reduced physical activity commuting to and at work, (3) longer commutes, (4) higher stress, (5) less sleep, (6) more machine and less human time, (7) higher rates of mental health disorders, (8) increased prescription and over-the-counter drug use, many of which increase appetite, and (9) higher salt intake.” There shouldn't be any citations there.

In the published article, there was an error in **Discussion**, *The Changing American Diet: History and Influence*, paragraph 6. The citation number (97) was incorrectly referenced as “Panuganti KK Nguyen M Kshirsagar RK. Obesity. In: StatPearls [Internet]. Treasure Island, FL: StatPearls Publishing (2021). Available online at: https://www.ncbi.nlm.nih.gov/books/NBK459357/.” The correct citation number is (94) and the correct reference should have been written as, “Panuganti KK, Nguyen M, Kshirsagar RK. Obesity. In: StatPearls [Internet]. Treasure Island, FL: StatPearls Publishing; 2021. Available from: https://www.ncbi.nlm.nih.gov/books/NBK459357/.”

In the published article, there was an error in **Discussion**, *The Changing American Diet: History and Influence*, paragraph 7. The citation numbers (9, 10, 98–100) were incorrectly referenced as “Walker AR Walker BF. The Surgeon General's Report on Nutrition and Health. Washington, DC: Government Printing Office; Department of Human Services (1989); National Research Council. Diet, Nutrition, and Cancer. Washington, DC: The National Academies Press (1982); Mayo Clinic,. Obesity. (2019). Available online at: https://www.mayoclinic.org/diseases-conditions/obesity/symptoms-causes/syc-20375742 (accessed July 21, 2021); Campbell TC Campbell TM. The China Study.Dallas, TX: Ben Bella (2006); Lee DS Chiu M Manuel DG Tu K Wang X Austin PC et al. Trends in risk factors for cardiovascular disease in Canada: temporal, socio-demographic and geographic factors. CMAJ. (2009) 181:55–66. doi: 10.1503/cmaj.08162919620271.” The correct citation numbers are (12, 13, 95–97) and the correct references should have been written as, “Walker AR, Walker BF. The Surgeon General's report on nutrition and health. Am J Clin Nutr. 1989 Oct;50(4):884–6. doi: 10.1093/ajcn/50.4.884; National Research Council (US) Committee on Diet, Nutrition, and Cancer. Diet, nutrition, and cancer. Washington, DC: National Academies Press (US); 1982. doi: 10.17226/371; Mayo Clinic. Obesity. 2019. Available from: https://www.mayoclinic.org/diseases-conditions/obesity/symptoms-causes/syc-20375742. Accessed 2021 Jul 6; Campbell TC, Campbell TM. The China Study. Dallas (TX): BenBella Books; 2006; and Lee DS, Chiu M, Manuel DG, Tu K, Wang X, Austin PC, et al. Trends in risk factors for cardiovascular disease in Canada: temporal, socio-demographic and geographic factors. CMAJ. 2009 Aug 4;181(3, 4):E55-66. doi: 10.1503/cmaj.081629.”

In the published article, there was an error in **Discussion**, *The Changing American Diet: History and Influence*, paragraph 7. The citation numbers (90, 101–109) were incorrectly referenced as “Dietary goals for the United States: statement of The American Medical Association to the Select Committee on Nutrition and Human Needs United States Senate. R I Med J. (1977) 60:576–81; Sims EAH Danforth E. Expenditure and storage of energy in man. J Clin Invest. (1987) 79:1019–25. doi: 10.1172/JCI1129133549776; Sims EAH Bray GA Danforth E. Experimental obesity in man. VI: The effect of variations in intake of carbohydrate on carbohydrate, lipid, and cortisol metabolism. Horm Metab Res. (1974) 6:70–7.4423813; Sims EAH Danforth E Horton ES. Endocrine and metabolic effects of experimental obesity in man. Recent Prog Horm Res. (1973) 29:457–96. doi: 10.1016/B978-0-12-571129-6.50016-64750591; Stene J Roberts I. A nutrition study on an indian reservation. J Am Diet Assoc. (1928) 3:215–22. doi: 10.1016/S0002-8223(21)35352-4; Richards RC DeCasseres M. The Problem of Obesity in Developing Countries: Its Prevalence Morbidity. BurlandWLSamuelPDYudkinJeditors (1974). p. 74–84. Available online at: http://pesquisa.bvsalud.org/portal/resource/es/med-9393; Prior IAM. The price of civilization. Nutr Today. (1971) 6:2–11. doi: 10.1097/00017285-197107000-00001; Moffatt RJ Sady SP Owen GM. Height, weight and skinfold thickness of Michigan adults. Am J Public Health. (1980) 70:1290–2. doi: 10.2105/AJPH.70.12.12907435748; Pennington AW. A reorientation on obesity. N Engl J Med. (1950) 248:959–64. doi: 10.1056/NEJM19530604248230113046654; and Castelli WP Anderson K Wilson PWF Levy D. Lipids and risk of coronary heart disease The Framingham Study. Ann Epidemiol. (1992) 2:23–8. doi: 10.1016/1047-2797(92)90033-M1342260.” The correct citation numbers are (26, 27, 98–105) and the correct references should have been written as, “Sims EA, Bray GA, Danforth E Jr, Glennon JA, Horton ES, Salans LB, O'Connell M. Experimental obesity in man. VI. The effect of variations in intake of carbohydrate on carbohydrate, lipid, and cortisol metabolism. Horm Metab Res. 1974;Suppl 4:70–7;Sims EA, Danforth E Jr, Horton ES, Bray GA, Glennon JA, Salans LB. Endocrine and metabolic effects of experimental obesity in man. Recent Prog Horm Res. 1973;29:457–96. doi: 10.1016/B978-0-12-571129-6.50016-6; American Medical Association. Dietary goals for the United States: statement of the American Medical Association to the Select Committee on Nutrition and Human Needs United States Senate. R I Med J. 1977;60:576–81. Available from: https://www.govinfo.gov/content/pkg/CPRT-95SPRT98364O/pdf/CPRT-95SPRT98364O.pdf; Sims EA, Danforth E Jr. Expenditure and storage of energy in man. J Clin Invest. 1987 Apr;79(4):1019–25. doi: 10.1172/JCI112913; Stene JA, Roberts LJ. A nutrition study on an Indian reservation. J Am Diet Assoc. 1928;3(4):215–22. doi: 10.1016/S0002-8223(21)35352-4; Richards RC, DeCasseres M. The problem of obesity in developing countries: its prevalence morbidity. In: Burland WL, Samuel PD, Yudkin J, editors. Obesity symposium: proceedings of a Servier Research Institute symposium held in December 1973. Edinburgh: Churchill Livingstone; 1974. p. 74–84; Prior IA. The price of civilization. Nutr Today. 1971 Jul;6(4):2–11; Moffatt RJ, Sady SP, Owen GM. Height, weight and skinfold thickness of Michigan adults. Am J Public Health. 1980 Dec;70(12):1290–2. doi: 10.2105/AJPH.70.12.1290; Pennington AW. A reorientation on obesity. N Engl J Med. 1953 Jun 4;248(23):959-64. doi: 10.1056/NEJM195306042482301; Castelli WP, Anderson K, Wilson PW, Levy D. Lipids and risk of coronary heart disease. The Framingham Study. Ann Epidemiol. 1992 Jan-Mar;2(1, 2):23-8. doi: 10.1016/1047-2797(92)90033-m.”

In the published article, there was an error in **Discussion**, *The Changing American Diet: History and Influence*, paragraph 7. The citation number (110) was incorrectly referenced as “Roberts CK Hevener AL Barnard RJ. Metabolic syndrome and insulin resistance: underlying causes and modification by exercise training. Compr Physiol. (2013) 3:1–58. 10.1002/cphy.c11006223720280.” The correct citation number is (106) and the correct reference should have been written as, “Roberts CK, Hevener AL, Barnard RJ. Metabolic syndrome and insulin resistance: underlying causes and modification by exercise training. Compr Physiol. 2013 Jan;3(1):1-58. doi: 10.1002/cphy.c110062.”

In the published article, there was an error in **Discussion**, *The Changing American Diet: History and Influence*, paragraph 8. The citation number (78) was incorrectly cited at the end of this sentence, “There was a >30% increase in overweight Americans from 1976–1980 (25.4%) to 1988–1991 (33.3%), associated with an 11% decrease in percent of fat calories (41.0–36.6%), a 4% decrease in daily calories (1,854–1,785 kcal), and a 9.8-fold increase in high fructose corn syrup.” There should not be any citations there.

In the published article, there was an error in **Discussion**, *The Changing American Diet: History and Influence*, paragraph 8. The citation number (80) was incorrectly referenced as, “Heini AF Weinsier RL. Divergent trends in obesity and fat intake patterns: the American paradox. Am J Med. (1997) 102:259–64. doi: 10.1016/S0002-9343(96)00456-19217594.” The correct citation number is (78) and the correct reference should have been written as, “World Health Organization. Diet, nutrition, and the prevention of chronic diseases: report of a joint WHO/FAO expert consultation. Geneva: World Health Organization; 2003. Available from: https://iris.who.int/bitstream/handle/10665/42665/WHO_TRS_916.pdf?sequence=1.”

In the published article, there was an error in **Discussion**, *The Changing American Diet: History and Influence*, paragraph 8. The citation number (88) was incorrectly referenced as, “Howard B V Van Horn L Hsia J Manson JAE Stefanick ML Wassertheil-Smoller S et al. Low-fat dietary pattern and risk of cardiovascular disease: the Women's Health Initiative randomized controlled dietary modification trial. J Am Med Assoc. (2006) 295:655–66. doi: 10.1001/jama.295.6.65518377781.” The correct citation number is (30) and the correct reference should have been written as, “Howard BV, Van Horn L, Hsia J, Manson JE, Stefanick ML, Wassertheil-Smoller S, et al. Low-fat dietary pattern and risk of cardiovascular disease: the Women's Health Initiative Randomized Controlled Dietary Modification Trial. JAMA. 2006 Feb 8;295(6):655–66. doi: 10.1001/jama.295.6.655.”

In the published article, there was an error in **Discussion**, *The Changing American Diet: History and Influence*, paragraph 8. The citation number (104, 105, 107, 111) were incorrectly referenced as, “Stene J Roberts I. A nutrition study on an indian reservation. J Am Diet Assoc. (1928) 3:215–22. doi: 10.1016/S0002-8223(21)35352-4; Richards RC DeCasseres M. The Problem of Obesity in Developing Countries: Its Prevalence Morbidity. BurlandWLSamuelPDYudkinJeditors (1974). p. 74–84. Available online at: http://pesquisa.bvsalud.org/portal/resource/es/med-9393; Moffatt RJ Sady SP Owen GM. Height, weight and skinfold thickness of Michigan adults. Am J Public Health. (1980) 70:1290–2. doi: 10.2105/AJPH.70.12.12907435748; and Taubes G. Good Calories, Bad Calories. New York, NY: Anchor Books (2008).” The correct citation number are (100, 101, 103, 107) and the correct references should have been written as, “Stene JA, Roberts LJ. A nutrition study on an Indian reservation. J Am Diet Assoc. 1928;3(4):215–22. doi: 10.1016/S0002-8223(21)35352-4; Richards RC, DeCasseres M. The problem of obesity in developing countries: its prevalence morbidity. In: Burland WL, Samuel PD, Yudkin J, editors. Obesity symposium: proceedings of a Servier Research Institute symposium held in December 1973. Edinburgh: Churchill Livingstone; 1974. p. 74–84; Moffatt RJ, Sady SP, Owen GM. Height, weight and skinfold thickness of Michigan adults. Am J Public Health. 1980 Dec;70(12):1290–2. doi: 10.2105/AJPH.70.12.1290; and Taubes G. Good Calories, Bad Calories. New York, NY: Anchor Books; 2008.”

In the published article, there was an error in **Discussion**, *The Changing American Diet: History and Influence*, paragraph 8. The citation number (112–115) were incorrectly referenced as, “Benedict FG Goodall HW Ash JE Langfeld HS Kendall AI Higgins HL. A Study of Prolonged Fasting. Washington, DC: Carnegie Institution for Science (2021); Talbot F. Severe infantile malnutrition: the energy metabolism with the report of a new series of cases. Am J Dis Child. (1921) 22:358–70. doi: 10.1001/archpedi.1921.0412004003300325996397; Benedict FG. Metabolism during starvation and undernutrition. N Y Med J. (1922) 115:249–56 and Fleming GB. An investigation into the metabolism in infantile atrophy, with special reference to the respiratory exchange. QJM. (1921) 14:171–86. doi: 10.1093/qjmed/os-14.54.171.” The correct citation numbers are (108–111) and the correct references should have been written as, “Benedict FG, Goodall HW, Ash JE, Langfeld HS, Kendall AI, Higgins HL. A Study of Prolonged Fasting. Washington, DC: Carnegie Institution for Science; 2021; Talbot FB. Severe infantile malnutrition: the energy metabolism with the report of a new series of cases. Am J Dis Child. 1921;22(4):358-70. doi: 10.1001/archpedi.1921.04120040033003; Benedict FG. Metabolism during starvation and undernutrition. N Y Med J. 1922;115:249–56; and Fleming GB. An investigation into the metabolism in infantile atrophy, with special reference to the respiratory exchange. QJM. 1921 Jan;os-14(54):171–86. doi: 10.1093/qjmed/os-14.54.171.”

In the published article, there was an error in **Discussion**, *The Changing American Diet: History and Influence*, paragraph 9. The citation number (116–119) were incorrectly referenced as, “Nestel P. A society in transition: developmental and seasonal influences on the nutrition of Maasai women and children. Food Nutr Bull. (1986) 8:2–18. doi: 10.1177/156482658600800107; Zhang J. Germline mutations in predisposition genes in pediatric cancer. N Engl J Med. (2016) 374:1390–1. doi: 10.1056/NEJMc160033827050225; Cleave TL. The neglect of natural principles in current medical practice. J R Nav Med Serv. (1956) 42:54–83. doi: 10.1136/jrnms-42-5513346756; and Campbell GD. Diabetes in Asians and Africans in and around durban. S Afr Med J. (1963) 37:1195–208.14078559.” The correct citation numbers are (112–115) and the correct references should have been written as “Nestel P. A society in transition: developmental and seasonal influences on the nutrition of Maasai women and children. Food Nutr Bull. 1986 Mar;8(1):1-23. doi: 10.1177/156482658600800107; Zhang J, Nichols KE, Downing JR. Germline mutations in predisposition genes in pediatric cancer. N Engl J Med. 2016 Apr 7;374(14):1391. doi: 10.1056/NEJMc1600338; Cleave TL. The neglect of natural principles in current medical practice. J R Nav Med Serv. 1956;42:55-83. doi: 10.1136/jrnms-42-55; and Campbell GD. Diabetes in Asians and Africans in and around Durban. S Afr Med J. 1963 Nov 30;37:1195-208.”

In the published article, there was an error in **Discussion**, *The Changing American Diet: History and Influence*, paragraph 9. The citation number (120–124) were incorrectly referenced as, “Hansen AW Christensen DL Larsson MW Eis J Christensen T Friis H et al. Dietary patterns, food and macronutrient intakes among adults in three ethnic groups in rural Kenya. Public Health Nutr. (2011) 14:1671–9. doi: 10.1017/S136898001000378221299918; Harding W. The diet of Tokelau island migrants in New Zealand. In: Migration and Health in New Zealand and the Pacific. Wellington: Wellington Hospital Epidemiology Unit (1977). pp. 113–116; Adachi H Hino A. Trends in nutritional intake and serum cholesterol levels over 40 years in Tanushimaru, Japanese men. J Epidemiol. (2005) 15:85–9. doi: 10.2188/jea.15.8515930804; Mailer G Nicola H. Decolonizing the diet: synthesizing Native-American history, immunology, and nutritional science. J Evol Heal. (2013) 1:7. doi: 10.15310/2334-3591.1014; and Li D. Effects of macronutrient distribution on weight and related cardiometabolic profile in healthy non-obese chinese: a 6-month, randomized controlled-feeding trial. EBioMedicine. (2017) 22:200–7. 10.1016/j.ebiom.2017.06.01728939486.” The correct citation numbers are (116–120) and the correct references should have been written as, “Hansen AW, Christensen DL, Larsson MW, Eis J, Christensen T, Friis H, et al. Dietary patterns, food and macronutrient intakes among adults in three ethnic groups in rural Kenya. Public Health Nutr. 2011 Sep;14(9):1671-9. doi: 10.1017/S1368980010003782; Harding WR, Russell CE, Davidson F, Prior IAM. Dietary surveys from the Tokelau Island migrant study. Ecol Food Nutr. 1986;19(2):83–97. doi: 10.1080/03670244.1986.9990951; Adachi H, Hino A. Trends in nutritional intake and serum cholesterol levels over 40 years in Tanushimaru, Japanese men. J Epidemiol. 2005 May;15(3):85–9. doi: 10.2188/jea.15.85; Mailer G. Decolonizing the diet: synthesizing Native-American history, immunology, and nutritional science. J Evol Health. 2013;1(1). doi: 10.15310/2334-3591.1014. Available from: https://escholarship.org/uc/item/83n957nb; and. Wan Y, Wang F, Yuan J, Li J, Jiang D, Zhang J, et al. Effects of macronutrient distribution on weight and related cardiometabolic profile in healthy non-obese Chinese: a 6-month, randomized controlled-feeding trial. EBioMedicine. 2017;22:200–7. doi: 10.1016/j.ebiom.2017.06.017.”

In the published article, there was an error in **Discussion**, *The Changing American Diet: History and Influence*, paragraph 9. The citation number (125) was incorrectly referenced as, “Marlowe FW Berbesque JC Wood B Crittenden A Porter C Mabulla A. Honey, Hadza, hunter-gatherers, and human evolution. J Hum Evol. (2014) 71:119–28. doi: 10.1016/j.jhevol.2014.03.00624746602.” The correct citation number is (121) and the correct reference should have been written as ”Marlowe FW, Berbesque JC, Wood B, Crittenden A, Porter C, Mabulla A, et al. Honey, Hadza, hunter-gatherers, and human evolution. J Hum Evol. 2014;71:119–28. doi: 10.1016/j.jhevol.2014.03.006.”

In the published article, there was an error in **Discussion**, *The Changing American Diet: History and Influence*, paragraph 9. The citation number (117, 126–130) were incorrectly referenced as, “Zhang J. Germline mutations in predisposition genes in pediatric cancer. N Engl J Med. (2016) 374:1390–1. doi: 10.1056/NEJMc160033827050225; Hoffman FL. Cancer and Diet.Baltimore, MD: Williams & Wilkins (1937); Cleave TL. Over-consumption, now the most dangerous cause of disease in westernized countries. Public Health. (1977) 91:127–31. doi: 10.1016/S0033-3506(77)80016-4882627; Burkitt DP. Some neglected leads to cancer causation. Natl Cancer Inst Monogr. (1979) 52:5–12; Hoffman FL. The Mortality From Cancer Throughout the World. Newark, NJ: Prudential Press (1915); and Christensen DL Eis J Hansen AW Larsson MW Mwaniki DL Kilonzo B et al. Obesity and regional fat distribution in Kenyan populations: impact of ethnicity and urbanization. Ann Hum Biol. (2008) 35:232–49. doi: 10.1080/0301446080194987018428015.” The correct citation numbers are (113, 122–126) and the correct references should have been written as, “Zhang J, Nichols KE, Downing JR. Germline mutations in predisposition genes in pediatric cancer. N Engl J Med. 2016 Apr 7;374(14):1391. doi: 10.1056/NEJMc1600338; Hoffman FL. Cancer and diet: with facts and observations on related subjects. Baltimore: Williams & Wilkins; 1937; Cleave TL. Over-consumption, now the most dangerous cause of disease in westernized countries. Public Health. 1977 May;91(3):127–31. doi: 10.1016/s0033-3506(77)80016-4; Burkitt DP. Some neglected leads to cancer causation. J Natl Cancer Inst. 1971 Nov;47(5):913–9; Hoffman FL. The mortality from cancer throughout the world. Newark, NJ: The Prudential Press; 1915; and Christensen DL, Eis J, Hansen AW, Larsson MW, Mwaniki DL, Kilonzo B, et al. Obesity and regional fat distribution in Kenyan populations: impact of ethnicity and urbanization. Ann Hum Biol. (2008) 35:232–49. doi: 10.1080/03014460801949870.”

The original article has been updated.
